# Multi-Omics Identification of Biomarkers for High-Altitude Pulmonary Hypertension

**DOI:** 10.3390/jcdd13050195

**Published:** 2026-04-30

**Authors:** Zhe Chen, Linhong Pang, Yidan Zheng, Li Xu, Mingjing Tang, Ziwen Zhao, Tianyu Wang, Jin Li, Yunfei Zhou, Lin Duo, Wenlong Zhu, Zhiling Luo, Fei Li, Da Zhu

**Affiliations:** 1 Department of Cardiovascular Surgery, Union Hospital, Tongji Medical College, Huazhong University of Science and Technology, 1277 Jiefang Ave, Wuhan 430022, China; cz09150806@163.com (Z.C.); u201910272@hust.edu.cn (Y.Z.); whunionxl@163.com (L.X.); 2 Department of Structural Heart Center, Fuwai Yunnan Cardiovascular Hospital, Affiliated Cardiovascular Hospital of Kunming Medical University, 528 Shahebei Rd, Kunming 650102, China; plh2835@163.com (L.P.); candysabrina@126.com (M.T.); zhaoziwen0214@163.com (Z.Z.); duolin@hotmail.com (L.D.); zhuwenlong@kmmu.edu.cn (W.Z.); 3 School of Health Policy and Management, Chinese Academy of Medical Sciences & Peking Union Medical College, Beijing 100730, China; 4 Department of Echocardiogram, Fuwai Yunnan Hospital, Affiliated Cardiovascular Hospital of Kunming Medical University, Kunming 650102, China; wangtianyu0730@126.com (T.W.); r1998lj@126.com (J.L.); colorfly1@126.com (Y.Z.); luozhiling@kmmu.edu.cn (Z.L.)

**Keywords:** HAPH, SERPINE2, proteomics, metabolomic

## Abstract

(1) Aim: The incidence of high-altitude pulmonary hypertension (HAPH) has risen in recent years and is expected to continue increasing; however, its diagnosis remains challenging. In this study, we employed proteomics and metabolomics to identify the proteins and metabolic biomarkers that contribute to the development of HAPH. (2) Methods: We applied integrated proteomics and metabolomics to match blood samples from 40 HAPH patients and 40 healthy controls in Yunnan’s high-altitude regions to characterize molecular profiles, identify biomarkers, and develop a predictive model. (3) Results: Proteomic analysis identified four proteins (A2IPH7, K1C14, PSME2, SERPINE2) commonly dysregulated in HAPH patients from two high-altitude regions. SERPINE2 was notably downregulated and showed a negative correlation with clinical severity, which was further validated in HAPH rat lung tissues and supported by UK Biobank data for idiopathic PAH. Concurrent metabolomics uncovered 11 shared metabolites, largely acyl fatty acids, enriched in pathways such as unsaturated fatty acid synthesis. Integration of these multi-omics data enabled the development of a robust predictive model. (4) Conclusion: Our study identified key protein and metabolic biomarkers involved in HAPH development, which were validated in animal models. Based on these findings, a predictive model was developed, highlighting SERPINE2 and 11 metabolites as promising targets for the prediction and prevention of HAPH.

## 1. Introduction

Pulmonary arterial hypertension (PAH) is characterized by severe pulmonary arterial hypertension, accompanied by pulmonary vascular remodeling and a gradual increase in pulmonary vascular resistance [1,2,3]. It is defined as a mean pulmonary arterial pressure greater than 20 mmHg at rest, measured through cardiac catheterization [3,4]. The World Health Organization (WHO) classifies PAH into five categories based on pathophysiological mechanisms, clinical manifestations, hemodynamic characteristics, and treatment management: PAH associated with left heart disease, PAH associated with pulmonary diseases and/or hypoxia, PAH associated with pulmonary artery obstruction, and PAH of unknown mechanisms and/or multifactorial origin [5,6,7]. Pulmonary arterial hypertension (PAH) specifically refers to Group 1 of this classification, whereas all other groups are collectively referred to as pulmonary hypertension (PH).

High-altitude pulmonary hypertension (HAPH) is classified as the third category in the above classification. In high-altitude regions such as the Tibetan Plateau in China, the Kyrgyzstan Plateau, and Ethiopia, where millions of people live above 2500 m, HAPH poses a significant public health burden [5,8]. The pathogenesis of HAPH is intricate. Research has demonstrated that factors such as hypoxia, cardiovascular diseases, and nitric oxide (NO) contribute to the progression of HAPH [9,10]. Due to the non-specific symptoms of HAPH patients and the limitations of routine diagnostic tests, an accurate diagnosis requires the exclusion of other forms of PAH [3], which presents numerous challenges in both diagnosis and treatment.

In recent years, proteomics and metabolomics have been widely applied to explore the pathogenesis of PAH and potential biomarkers. Previous studies have utilized metabolomics to investigate PAH and identified significant differences in amino acid and lipid metabolites between PAH patients and healthy controls [11,12,13]. Other studies using proteomics have suggested that proteins such as SULT1A1 (sulfotransferase 1A1) and TSP2 (thrombospondin-2) may serve as potential biomarkers involved in the pathogenesis of HAPH [14,15]. However, few studies have employed an integrated multi-omics approach to thoroughly investigate the pathogenesis and biomarkers of HAPH.

In this study, we established a rigorously matched cohort and performed integrated proteomic and metabolomic analyses on blood samples from 80 pairs of participants from Yunnan Province, China. The participants, including individuals from Diqing Tibetan Autonomous Prefecture (average altitude: 3262 m) and Lijiang City (average altitude: 2932 m), represented various minority ethnic groups such as the Naxi and Tibetan populations. The cohort included an equal number of control subjects and HAPH patients, aiming to describe the proteomic and metabolomic profiles of HAPH patients, explore and identify potential molecular targets and biomarkers for HAPH, and develop a robust predictive model based on omics data, which contributes to the advancement of early screening and personalized treatment.

## 2. Materials and Methods

### 2.1. Epidemiological Survey

#### 2.1.1. Survey Sites and Participants

This cardiovascular disease screening program was conducted at high altitudes (2500 m and above) in Yunnan Province, a low-income province in Southwest China. The study period was from September 2023 to January 2024, and the study participants were sampled from Lijiang City (mean altitude of 2932 m) and Diqing Tibetan Autonomous region (mean altitude of 3262 m) using a multi-stage stratified cluster sampling method. Firstly, all townships in Yunnan Province above 2500 m above sea level were divided into rural and urban, and 2 townships were selected in each of these two strata by a simple random sampling method. Secondly, 4 village committees continued to be randomly sampled from the 4 townships sampled above, with a total of 16 village committees. Finally, residents aged 35 years and above who have been living in the sampled village committees for more than 6 months were included as participants in the survey for this screening project. The survey locations are shown in Figure S1. The STROBE checklist is shown in Figure S4.

#### 2.1.2. Data Collection

A survey team of approximately 20 trained internists, sonographers, nurses, pharmacists, and volunteers performed questionnaires, physical examinations, echocardiographic measurements, and laboratory tests on the participants. First, participants’ general demographic characteristics (age, gender, ethnicity, education, marital status, occupation, income), behavioral lifestyle (smoking, alcohol consumption, diet), disease history, and medication history were collected using a standardized questionnaire. The participants were asked to sit still and rest for at least 5 min, and their systolic blood pressure (SBP) and diastolic blood pressure (DBP) were measured using an OMRON HBP-1300 electronic sphygmomanometer. Finally, experienced nurses collected blood samples from participants who had been fasting for more than 8 h. All blood samples were separated on site and stored cryogenically in −80° liquid nitrogen tanks for subsequent analysis. All procedures involving human participants were conducted in accordance with the ethical standards of the Declaration of Helsinki (1975, revised in 2013) and were approved by the Ethics Committee of Fuwai Yunnan Cardiovascular Hospital (NO. 2023-026-01). Informed consent was obtained from all individual participants included in the study.

#### 2.1.3. Echocardiography Assessment

Each participant’s transthoracic echocardiogram was conducted at the screening facility by two skilled and trained echocardiographers using a portable ultrasound device (Vivid™ iq, GE Medical Systems Trade and Development Co., Ltd., Shanghai, China) with Probe (M5Sc). The echocardiography device is equipped with M-mode, 2D, pulsed wave Doppler, continuous wave Doppler, and color flow Doppler modes. The structure, function, location, and number of heart valves, valve thickening, echo enhancement, calcification, and flow velocity of each valve were recorded. These ultrasound images were re-verified for diagnosis by a 3rd sonographer before statistical analysis of the data to ensure the accuracy of the diagnostic results. In this high-altitude community study, screening of PHT is based on echocardiography with reference to guidelines [3]. Doppler-derived tricuspid regurgitant velocities (TRV) were measured and the systolic right ventricle-to-atrium pressure gradient was estimated using the simplified Bernoulli’s equation. Pulmonary systolic pressure (SP) was estimated from a trans-tricuspid gradient calculated from the maximum velocity (V) of continuous Doppler tricuspid regurgitation, as 4 × V^2^ + 5 mmHg assigned to right atrial pressure. The cut-off valve for screening PH is TRV > 2.8 m/s and pulmonary SP > 36 mmHg. The first step in assessing the echocardiographic probability of PAH being present is to measure the peak TRV. If this is a good-quality signal and is greater than 3.4 m/s, there is a high probability of PAH being present. If the peak TRV ranges from 2.8 to 3.4 m/s, the probability of PAH is assessed in combination with other echocardiographic markers such as RV dilation. In the control group, TRV should be less than 2.8 m/s or not measurable and without other echocardiographic markers of PAH. This study is based on echocardiographic screening conducted in a high-altitude community. Although echocardiography is not the gold standard for diagnosing PAH, it is a readily available bedside technique that is non-invasive and recognized as the primary non-invasive method for assessing PAH [3,16,17].

#### 2.1.4. HAPH and Control Groups

The valid sample for the epidemiological survey was 2458 inhabitants, among whom 131 cases of pulmonary hypertension were diagnosed. Based on the following exclusion criteria, a total of 40 PAH individuals who had resided in the area for more than 10 years were identified as the case group, and 40 healthy adults residing at the same altitude level as the HAPH group were selected as the control group. The exclusion criteria were as follows: (1) have a history of drug or poison administration; (2) have congenital heart disease, heart failure, left heart valvular heart disease, pulmonary embolism or thrombosis, severe metabolic abnormalities, hematological disorders, or renal failure; (3) suffering from COPD; (4) suffering from lung tumor; (5) have a history of tuberculosis; (6) have autoimmune disease; (7) have infectious diseases such as rheumatic diseases, infective endocarditis, hepatitis B,C,E and other infectious diseases; (8) receiving medication for pulmonary hypertension; (9) cerebrovascular events (transient ischemia or stroke) within the last 3 months; (10) those with severe hypertension (>150/100 mmHg).

### 2.2. Quality Control of Genotype and Imputation Data

Genotypic data were imputed using the TOPMed R^2^ reference panel. Genetic data from the UK Biobank were phased using Eagle v2.4 and converted from GRCh37 to GRCh38 with LiftOver. Imputation was performed with Minimac4 v1.0.2 in 1Mb segments, referencing the TOPMed R^2^ panel. Markers with an imputation quality score (R^2^) below 0.1 were excluded, leaving 677,037 markers for analysis. The imputed VCF files were subsequently converted to BGEN format using qctool v2.0.8, with genotype probabilities stored at 8-bit precision. Additionally, a minimum allele frequency (MAF) threshold of 0.01 was applied for quality control purposes.

### 2.3. Ethics Statement

The study utilizing the UK Biobank (UKB) was approved by the North West Multicenter Research Ethics Committee (approval number: 11/NW/0382). All participants provided written informed consent prior to their inclusion in the study. Genotypic and clinical data were sourced from the UKB datasets in accordance with application number 105945.

All experimental animals were obtained from Jiangsu Youdu Biotechnology. All animal procedures were approved by the Animal Care and Use Committee of Tongji Medical College and complied with the European Union Directive 2010/63/EU on the protection of animals used for scientific purposes.

### 2.4. Genome-Wide Association Study Data

Summary of pan-ancestry genetic analysis of the UK Biobank was included in this study to avoid potential ignorance in a multi-ancestry study. Abdominal aortic aneurysm was defined as phenocode 442.11, including 1306 cases and 408,565 controls of both sexes. Detailed information can be found in:

https://docs.google.com/spreadsheets/d/1AeeADtT0U1AukliiNyiVzVRdLYPkTbruQSk38DeutU8/edit?gid=1450719288#gid=1450719288 (accessed on 6 June 2024).

The data for this study were sourced from various repositories. The eQTL data were obtained from the GTEx v8 dataset, with the tissue type being whole blood. For pQTL data, the source was the file available at:

https://ftp.ebi.ac.uk/pub/databases/gwas/summary_statistics/GCST90241001-GCST90242000/GCST90241257/harmonised/GCST90241257.h.tsv.gz (accessed on 6 June 2024).

Regarding the analysis parameters, for pQTL MR, the exposure was considered significant if the p-value was less than 1 × 10^−5^, with linkage disequilibrium (LD) R^2^ < 0.5. For eQTL MR, the exposure was considered significant with a *p*-value threshold of less than 1 × 10^−4^ and LD R^2^ < 0.9. All analyses were based on publicly available GWAS summary statistics that do not require special access permissions.

### 2.5. Data Source of MR Analysis

The exposure data for this study were derived from plasma metabolite profiling performed by Nightingale Health Plc. on baseline samples from 500,000 UK Biobank participants. Biomarkers were quantified using nuclear magnetic resonance (NMR) spectroscopy. The dataset includes 118,461 baseline EDTA plasma samples, along with approximately 4000 repeat assessment samples. Data collection and processing followed EN ISO 13485 standards [18], and biomarkers were measured using CE-marked In Vitro Diagnostic Medical Devices. In this study, key quality control parameters for the exposure data included a significance threshold of *p* < 0.05 to identify robust associations. For the outcome data, genotype and imputation data were processed using Plink2 software (version 2.00a6).

### 2.6. Mendelian Randomization Analysis and Sensitivity Analysis

Mendelian randomization (MR) analyses were conducted using the TwoSampleMR R package (version 0.6.4), with SERPINE2 expression level as the exposure and idiopathic PAH as the outcomes.

Instrumental variables (IVs) were chosen based on genome-wide significance to reduce confounding from variants that are directly linked to the outcome but not mediated by the exposure. To prevent the use of weak instruments, IVs with an F-statistic below 10 were excluded.

To investigate the causal relationship between SERPINE2 gene expression and HAPH, we selected common variants (minor allele frequency ≥ 0.01) associated with SERPINE2 gene expression from the Genotype-Tissue Expression (GTEx) project, Version 7. After excluding variants in high linkage disequilibrium, we performed Mendelian randomization, taking into account the linkage disequilibrium between the variants. Pleiotropy and heterogeneity of eQTL and pQTL are presented in Tables S1–S4.

### 2.7. Animal Experiments

Twenty adult male rats, aged 6–8 weeks, were randomly assigned to two groups. One group was housed in a pathogen-free, temperature-controlled environment with normal oxygen levels, while the other group was kept in a pathogen-free, temperature-controlled hypoxic environment (oxygen concentration of 10–12%) and fed a standard diet for 4–6 weeks. Afterward, the rats were anesthetized with 2.5% isoflurane, and pulmonary hypertension was assessed by measuring pulmonary artery pressure. At the study’s conclusion, euthanasia was performed via intraperitoneal injection of a lethal dose of sodium pentobarbital (100 mg/kg), and lung tissue samples were collected for further analysis. All animal procedures were approved by the Animal Care and Use Committee of Tongji Medical College and complied with the European Union Directive 2010/63/EU on the protection of animals used for scientific purposes. For humane euthanasia in rat hypoxia studies, sodium pentobarbital overdose (150–200 mg/kg, intraperitoneal) was administered, inducing rapid unconsciousness (<60 s) followed by respiratory and cardiac arrest within 5 min. Death was confirmed by absence of corneal reflex, fixed pupils, and no response to toe pinch, in compliance with AVMA guidelines (2020).

### 2.8. Immunofluorescence Staining

Immunofluorescence staining was performed to detect the expression of SERPINE2 and αSMA proteins in rat pulmonary vascular smooth muscle cells. Briefly, lung tissue sections from rats were washed twice with phosphate-buffered saline (PBS) and fixed in 4% paraformaldehyde for 10 min. The sections were then washed three times with PBS and blocked with Quick Block immunostaining blocking solution (Beyotime, P0260, Shanghai, China) for 15 min, followed by three additional PBS washes. The samples were incubated overnight at 4 °C with primary antibodies (Proteintech, 14395-1-AP, 66203-1-Ig, Wuhan, China), followed by incubation with fluorescence-conjugated secondary antibodies. DAPI (Sigma-Aldrich, St. Louis, MO, USA) was used for counterstaining. Images were captured using a Leica fluorescence microscope (Carl Zeiss, Jena, Germany) and analyzed with ImageJ software (version 1.53, National Institutes of Health, Kensington, MD, USA).

### 2.9. Sample Preparation

Blood samples were collected in tubes containing ethylenediaminetetraacetic acid (EDTA) or citrate as anticoagulants to prevent coagulation. Fresh blood samples were centrifuged at 1600 g for 10 min at 4 °C to separate the plasma from the blood cells, and the plasma supernatant was collected.

### 2.10. Proteomics Analysis

This study employs next-generation label-free quantitative proteomics for analysis. Using the Data-Independent Acquisition (DIA) mode, this method offers unmatched proteome coverage while ensuring precise, highly reproducible quantification of a large number of proteins across each sample. The DIA process consists of three essential steps: 1. Building the spectral library: the spectral library collects all detectable non-redundant, high-quality peptide information (MS/MS spectra) from the sample, which serves as the template for peptide identification during data analysis. This library includes data on the fragment ion intensities and retention times of peptide peaks. The spectral library is constructed from data acquired through Data-Dependent Acquisition (DDA) analysis of the samples of interest. 2. Acquiring data from large sample sets in DIA mode: Data-Independent Acquisition (DIA, also known as SWATH) mode uses high-resolution mass spectrometry to capture peptide ion features based on both mass-to-charge ratio (*m/z*) and retention time. 3. Data analysis: the DIA data are deconvoluted and integrated with the DDA spectral library to obtain qualitative and quantitative information for peptides and proteins. Differential analysis of the data is performed using the MSstats R package (version 4.10.1), which also analyzes the biological functions of differentially expressed proteins.

### 2.11. DIA Protein Quantification and Statistical Analysis

After extracting ion peak areas from the spectra generated in this experiment, the MSstats R package (version 4.10.1) is used for systematic error correction and normalization of each sample. A linear mixed-effects model is then applied to assess whether the differences in protein expression between the comparison groups are statistically significant. Differential proteins are considered significant if they meet the criteria of fold change > 2 and adj_*p*-value < 0.05.

The study used the MSstats R package (version 4.3.0) to assess the significant differences in proteins or peptides between different samples. The core algorithm of MSstats is the linear mixed-effects model, which is used for data preprocessing and significance testing based on comparison groups.

### 2.12. Differential Expression Clustering

Each cluster consists of two parts: the intersection and the union. The intersection requires proteins to show significant differences in all comparison groups, while the union requires significant differences in at least one comparison group.

In this study, Euclidean distance and hierarchical clustering methods were used to cluster the differentially expressed proteins.

### 2.13. Main Instruments and Reagents for Metabolomics

Main Instruments: Low-Speed High-Speed Centrifuge (Centrifuge 5430, Eppendorf, Eppendorf AG, Hamburg, Germany), Vortex Mixer (QL-901, Qilinbell Instrument Manufacturing Co., Ltd., Haimen, China), ultrapure water system (Milli-Q Integral, Millipore Corporation, Burlington, MA, USA), Freeze Vacuum Concentrator (Maxi Vacbeta, GENE COMPANY, Hong Kong, China), Tissue Homogenizer (JXFSTPRP, Shanghai Jingxin, Shanghai, China). Reagents: Methanol (A454-4) and Acetonitrile (A998-4), LCMS grade (Thermo Fisher Scientific, Waltham, MA, USA); ammonium formate (17843-250G, Honeywell Fluka, Seelze, Germany), formic acid (50144-50mL, DIMKA, Beijing, China), water from the ultrapure water system. Internal standards: d3-Leucine, 13C9-Phenylalanine, d5-Tryptophan, 13C3-Progesterone.

### 2.14. Metabolite Extraction

Thaw plasma samples (stored at −20 °C) at 4 °C until no ice remains. For each sample (including QC), take 100 µL and transfer it into an EP tube, then freeze the remaining sample. Add 700 µL of extraction solvent containing internal standard 1 (methanol: Acetonitrile: water = 4:2:1, *v*/*v*/*v*) to each sample. Shake the sample for 1 min and incubate at −20 °C for 2 h. Centrifuge at 25,000× g for 15 min at 4 °C. Carefully remove the supernatant and transfer 600 µL to a new EP tube. Dry the sample under nitrogen. Add 180 µL of methanol : water (1:1 *v*/*v*), vortex for 10 min until completely dissolved. Centrifuge again at 25,000× g for 15 min at 4 °C, then transfer the supernatant to a new EP tube.

### 2.15. UPLC-MS Analysis

This study used the Waters UPLC I-Class Plus (Waters, Milford, MA, USA) coupled with the Q Exactive High-Resolution Mass Spectrometer (Thermo Fisher Scientific, Waltham, MA, USA) for metabolite separation and detection. Chromatographic separation was performed using a BEH C18 column (1.7 μm, 2.1 × 100 mm, Waters, USA). For positive ion mode, the mobile phases consisted of A: water with 0.1% formic acid; and B: methanol with 0.1% formic acid. In negative ion mode, A was 10 mM ammonium formate in water, and B was 95% methanol with 10 mM ammonium formate. The gradient elution program was as follows: 0–1 min at 2% B, 1–9 min from 2% to 98% B, 9–12 min at 98% B, 12–12.1 min from 98% to 2% B, and 12.1–15 min at 2% B. The flow rate was 0.35 mL/min, the column temperature was set to 45 °C, and the injection volume was 5 µL. Mass spectrometry analysis was performed using the Q Exactive Mass Spectrometer (Thermo Fisher Scientific, Waltham, MA, USA) for both MS and MS/MS data acquisition. The mass range was set from 70 to 1050 *m/z*, with a resolution of 70,000 for MS and 17,500 for MS/MS. Automatic Gain Control (AGC) values were 3e6 for MS and 1e5 for MS/MS, with maximum injection times of 100 ms and 50 ms, respectively. The top 3 precursor ions were selected for fragmentation based on ion intensity, and MS/MS data were collected. Stepped normalized collision energy (NCE) was applied at 20, 40, and 60 eV. ESI ion source parameters included a sheath gas flow rate of 40, auxiliary gas flow rate of 10, spray voltage of 3.80 kV for positive mode and 3.20 kV for negative mode, capillary temperature of 320 °C, and auxiliary gas heater temperature of 350 °C.

### 2.16. Software and Parameters

The raw data from the mass spectrometer were imported into Compound Discoverer 3.3 (Thermo Fisher Scientific, Waltham, MA, USA) for analysis, utilizing databases such as BMDB (BGI Metabolome Database), mzCloud, and ChemSpider to identify metabolites. The data analysis parameters included a parent ion mass deviation of <5 ppm, a fragment ion mass deviation of < 10 ppm, and a retention time deviation of <0.2 min. The differential metabolites in this project were selected based on the following criteria: 1. VIP ≥ 1 in the OPLS-DA model; 2. fold change ≥ 1.2 or ≤0.83; and 3. *p*-value < 0.05.

### 2.17. Processing of Proteomic and Metabolomic Data

Data statistical analysis was performed using GraphPad Prism 10 software and R-studio scripts (version 4.3.0). After imputing missing values and normalizing the data for dimensionality reduction, Student’s *t*-test was used for significance analysis to identify differentially expressed proteins and metabolites between HAPH and healthy controls. Pearson correlation coefficients were used to test the correlation between proteins and metabolites and HAPH clinical indicators (such as tricuspid regurgitation velocity and pulmonary systolic pressure). In R, the correlation R and *p*-values between proteins, metabolites, and clinical indicators of HAPH were calculated. Bioinformatics analysis was performed using R-studio scripts. Only proteins with NA (missing values) excluded were used for differential expression analysis. In the volcano plot, proteins and metabolites with *p* < 0.05 and fold changes > 1.2 were marked as upregulated and downregulated, respectively. To further explore the functional characteristics of proteins and metabolites related to phenotypic changes, clustering heatmaps of protein and metabolite expression across different samples were generated using pheatmap (version 1.0.12) and mfuzz (version 2.52.0). GO and KEGG enrichment analysis of differentially expressed proteins and metabolites was conducted using the Phyper function based on hypergeometric tests, with a Q-value threshold of ≤0.05, and the results were defined as significantly enriched within the candidate proteins and metabolites.

### 2.18. Statistical Analyses

For the baseline characterization of the study subjects, data are presented as means and standard deviations for continuous variables and as numbers (percentages) for categorical variables. For the comparison of baseline characteristics between the HAPH and control groups, Chi-square or *t*-tests were used when appropriate.

## 3. Results

### 3.1. Cohort Characteristics

A total of 80 subjects were included in this study, including 36 subjects in the Diqing area (mean altitude of 3262 m) and 44 subjects in the Lijiang area (mean altitude of 2932 m), with a sample size of 1:1 for HAPH and control groups. The baseline character was comparable between HAPH and control group (Table 1). For participants from the Diqing area, the mean age of the HAPH and control groups was similar (64.3 ± 7.9 vs. 65.4 ± 10.0 years), with the majority being female (72.2% vs. 77.8%) and Tibetan (77.8% vs. 83.3%). No statistical differences between the two groups were observed between smoking, alcohol consumption, waist circumference, BMI, blood pressure, and various biochemical parameters (all *p* > 0.05). However, right ventricular end-diastolic diameter was significantly higher in the HAPH group than in the control group (*p* < 0.05). For study subjects from the Lijiang area, the mean age was similar in the HAPH and control groups (55.0 ± 10.4 vs. 55.3 ± 10.4 years), with a majority of females (90.9% vs. 90.9%) and Naxi ethnicity (63.6% vs. 77.3%). The SBP was significantly higher in the HAPH group than in the control group (129.8 ± 11.0 vs. 121.8 ± 12.9 mmHg); significantly higher right ventricular end-diastolic diameter was also noted in the HAPH group than in the control group (all *p* < 0.05). Typical echocardiographic images of two HAPH participants from Lijiang clearly demonstrated increased right ventricular dimensions, abnormal septal motion, and tricuspid regurgitation, as presented in Figure S5.

### 3.2. Proteomic Sequencing Results of HAPH Patients and Healthy Controls

**Figure 1:**
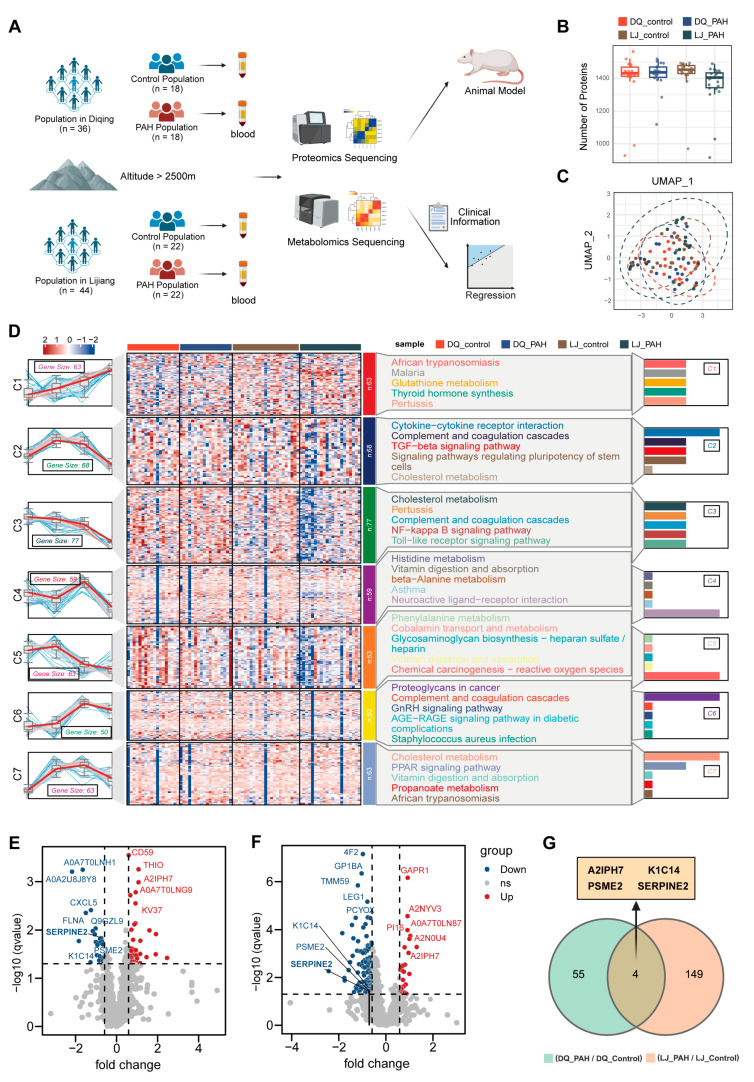
The cohort information and proteomic sequencing results of high-altitude pulmonary hypertension patients and healthy controls. (**A**) Flowchart of study (Diqing *n* = 36, Lijiang *n* = 44). (**B**) Proteome results identified the number of proteins obtained in two groups of samples. (**C**) The UMAP (Uniform Manifold Approximation and Projection) plot showing the differences between the two groups of samples. (**D**) Proteins were classified according to their expression in each group, Kyoto Encyclopedia of Genes and Genomes (KEGG) enrichment analysis of each cluster. (**E**,**F**) Volcano diagram showing upregulated and downregulated proteins in both regions. (**G**) Venn diagram showing that the common proteins were A2IPH7, K1C14, PSME2 and SERPINE2. PAH (Pulmonary arterial hypertension).

In this study, peripheral blood samples were collected from participants for proteomic and metabolomic analyses, followed by validation through animal experiments and the development of a predictive model. The flowchart is shown in Figure 1A. The proteomic results identified over 1400 proteins on average, with no significant difference in protein detection levels between the disease and control groups (Figure 1B). Further analysis revealed the UMAP dimensionality reduction results based on peripheral blood high-throughput proteomics data, showing a concentrated distribution of samples in both groups (Figure 1C). Based on the expression level variations in proteins across the two sample groups, the proteins were clustered into seven groups. Enrichment analysis using the Kyoto Encyclopedia of Genes and Genomes (KEGG) for each cluster indicated that amino acid metabolism and neuroactive ligand–receptor interactions were predominantly enriched in cluster 4, which showed conserved patterns between the two regions and a negative correlation with the disease (Figure 1D). Notably, other clusters also exhibited significant enrichments: cluster 2 was enriched in cytokine-related pathways, cluster 5 in reactive oxygen species pathways, and cluster 7 in cholesterol metabolism and PPAR signaling pathways, suggesting their potential involvement in the pathogenesis of high-altitude pulmonary hypertension. These findings highlight the multifaceted nature of HAPH, implicating not only metabolic dysregulation but also immune and oxidative stress responses in disease development. Volcano plots displayed the upregulated and downregulated proteins in HAPH patients from the Diqing and Lijiang regions (Figure 1E,F). A total of 59 differential proteins were identified in the Diqing region samples, while 153 differential proteins were found in the Lijiang region samples. Cross-model analysis of the Diqing and Lijiang HAPH samples revealed four common differential proteins (A2IPH7, K1C14, PSME2, SERPINE2) between the two regions (Figure 1G). These findings suggest that these proteins could serve as potential molecular biomarkers for HAPH, providing important insights for further research into their diagnostic and therapeutic value.

### 3.3. Differences in the Proteome Between HAPH Patients and Healthy Controls

**Figure 2:**
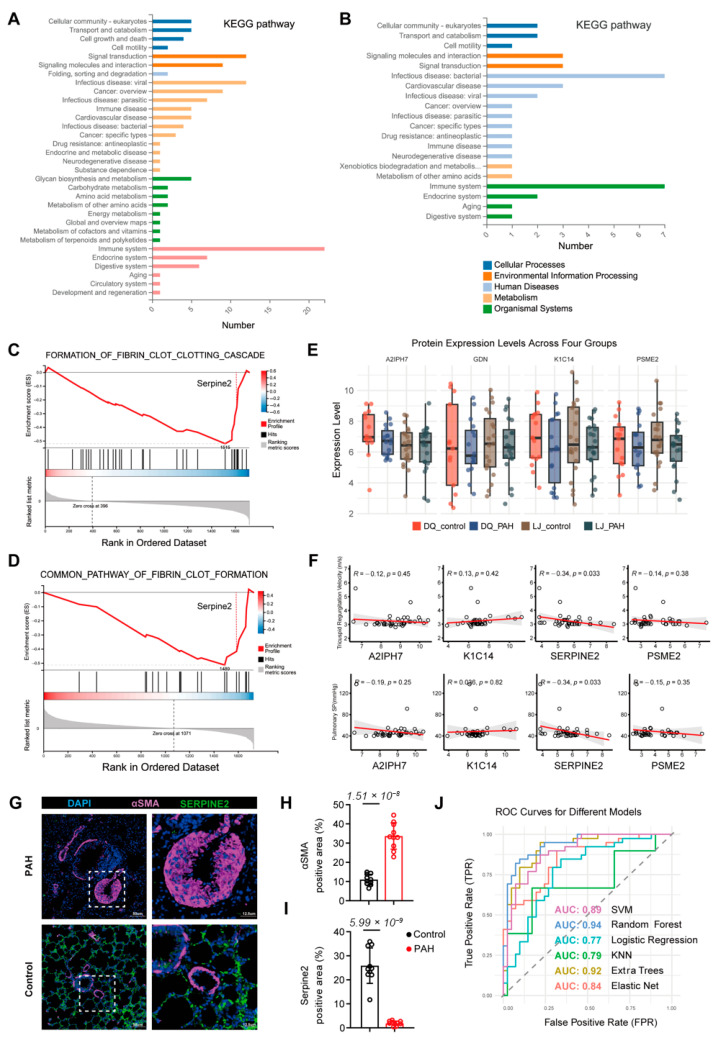
Proteomic sequencing results revealed differences in disease and healthy groups between the two regions. (**A**,**B**) Kyoto Encyclopedia of Genes and Genomes (KEGG) enrichment shows differential protein enrichment of pathways between the two regions. (**C**,**D**) GSEA results show that fibrin clot formation is a significant downregulated pathway at both regions. (**E**) Expression of four shared proteins in two groups of samples. (**F**) Scatter plot showing correlation of four proteins with clinical indicators of HAPH. (**G**–**I**) Lung sections suggest that SERPINE2 is significantly downregulated in plateau rats with HAPH (*n* = 10). (**J**) Survival curves of HAPH under different predictive models based on proteomics. PAH (Pulmonary arterial hypertension).

To better understand the functions of different proteins in the pathogenesis of HAPH, we further investigated the differentially expressed proteins (DEPs) and enriched pathways between the case and control groups. As shown in the results, the differential protein expression profiles in the Diqing and Lijiang populations were significantly enriched in pathways associated with cell processes, environmental information processing, human diseases, metabolism, and biological systems (Figure 2A,B). Gene Set Enrichment Analysis (GSEA) confirmed these findings, with fibrin clot formation emerging as the most significantly downregulated GSEA pathway in both regions, and SERPINE2 showing significant changes within the pathway (Figure 2C,D). In the two groups of control and HAPH samples, SERPINE2 (GDN), K1C14, and PSME2 were significantly downregulated in the HAPH group (Figure 2E). Scatter plots demonstrated a negative correlation between SERPINE2 and TRV as well as pulmonary systolic pressure (Figure 2F). Further immunofluorescence analysis confirmed that, compared to the normal group, lung tissue sections from hypoxia-induced high-altitude pulmonary hypertension rats exhibited a significant upregulation of the vascular smooth muscle cell marker αSMA and a marked downregulation of the SERPINE2 protein (Figure 2G–I). Subsequently, the study established multiple predictive models for HAPH based on proteomics data, with the Random Forest model achieving an area under the curve (AUC) of 0.94 (Figure 2J), demonstrating high predictive capability for HAPH occurrence.

### 3.4. Identification of SNPs Associated with PAH Progression Through the UK Biobank

**Figure 3:**
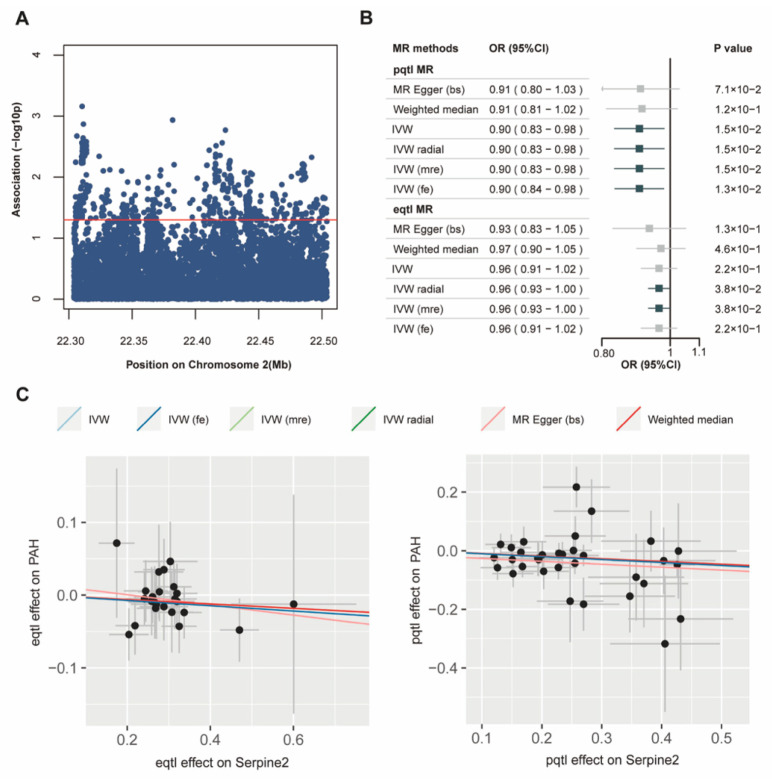
UK Biobank demonstrated the association between SERPINE2 and idiopathic Pulmonary arterial hypertension (PAH). (**A**) Manhattan plots of idiopathic PAH in the UK Biobank (PAH *n* = 787, normal *n* = 486,880). The red horizontal lines showed the suggestive genome-wide significance threshold (*p* < 0.05). (**B**) Forest plots demonstrating the negative correlation between SERPINE2 and idiopathic PAH. (**C**) Scatter plot illustrating the effect of SERPINE2 on idiopathic PAH with six MR methods.

To uncover the genetic basis associated with the development of PAH, we utilized data from the UK Biobank and selected a cohort of patients diagnosed with idiopathic PAH. Based on the progression during the follow-up period, the cohort was divided into two groups: the control group and the PAH group. We then established a strict threshold to filter and identify SNPs closely related to PAH (Figure 3A). The SNP loci table of eQTL and pQTL is provided in Tables S5 and S6. Our analysis revealed a consistent negative correlation between the SERPINE2 protein and PAH. Specifically, at the protein level, although no significance was observed with the MR Egger method (OR = 0.91, 95%CI = 0.80–1.03) and the weighted median method (OR = 0.91, 95%CI = 0.81–1.02), significant differences were observed using the IVW method (OR = 0.90, 95%CI = 0.83–0.98), IVW radial method (OR = 0.90, 95%CI = 0.83–0.98), IVW (mre) method (OR = 0.90, 95%CI = 0.83–0.98), and IVW (fe) method (OR = 0.90, 95%CI = 0.84–0.98). The SERPINE2 gene was validated using the IVW radial method (OR = 0.96, 95%CI = 0.93–1.00) and the IVW (mre) method (OR = 0.96, 95%CI = 0.93–1.00) (Figure 3B). Other results related to eQTL and pQTL MR are presented in Figures S2 and S3. The forest plot data for eQTL and pQTL analyses are derived from the respective datasets (Tables S7 and S8). eQTL and pQTL analyses jointly suggested a significant negative correlation between plasma SERPINE2 and PAH (Figure 3C).

### 3.5. Metabolomic Sequencing Results of HAPH Patients and Healthy Controls

**Figure 4:**
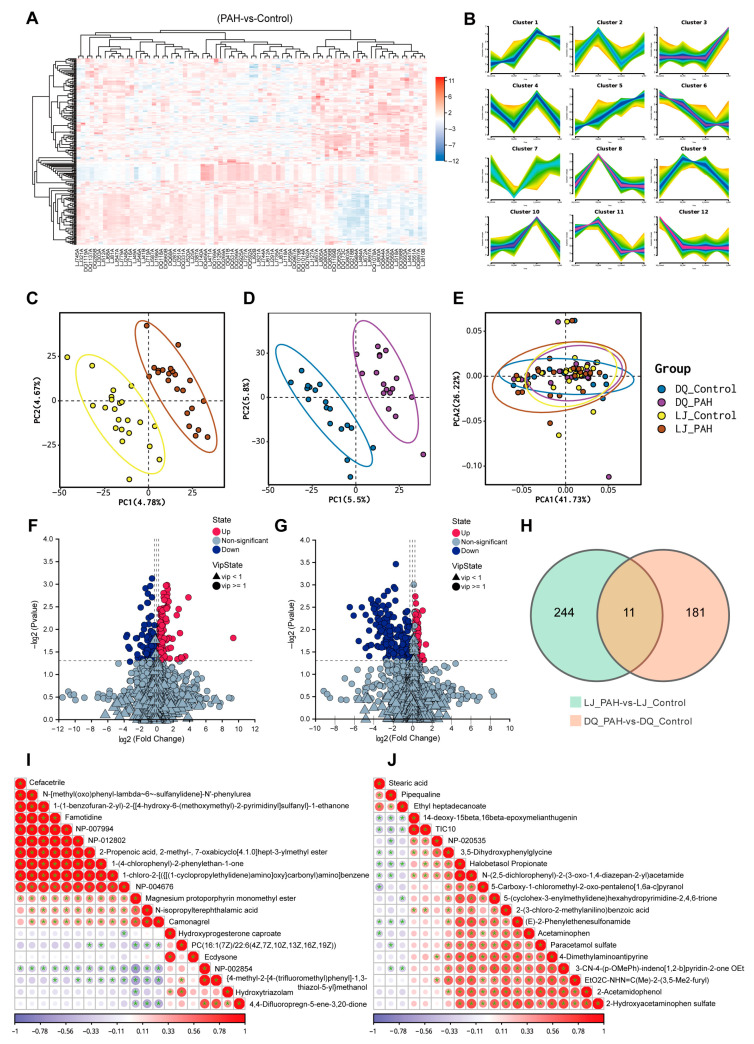
Metabolomic sequencing results of high-altitude pulmonary hypertension patients and healthy controls. (**A**) Heatmap demonstrating metabolite differences between Pulmonary arterial hypertension (PAH) and controls (Diqing *n* = 36, Lijiang *n* = 44). (**B**) Differential metabolites between the two regions were categorized into 12 clusters based on the level of metabolite change. (**C**–**E**) PCA dimensionality reduction and OPLSDA dimensionality reduction results. (**F**,**G**) Volcano diagram showing upregulated and downregulated metabolites in both regions. (**H**) Venn diagram reveals 11 metabolites as common metabolites between the two regions. (**I**,**J**) The correlation matrix shows the pairwise relationships between 11 metabolites in both regions.

Studies have shown that multiple metabolic pathways are altered in the pathogenesis of PAH [13]. To further investigate the changes in metabolite levels in the blood of HAPH patients, we conducted metabolomic analysis of blood samples from both patients and high-altitude healthy controls (Figure 4A). Based on the metabolic level changes in metabolites in the control and patient groups from both the Diqing and Lijiang regions, the differential metabolites were categorized into 12 distinct clusters, with clusters 2, 4, and 11 showing conserved patterns between the two regions (Figure 4B). Principal component analysis (PCA) of the blood samples from both regions showed no significant difference in metabolite detection between the control and patient groups, with similar PCA distribution. However, OPLSDA revealed significant differences (Figure 4C–E). Volcano plots display the upregulated and downregulated metabolites in HAPH patients from the Diqing and Lijiang regions (Figure 4F,G). A total of 255 metabolites were identified in Diqing samples, while 192 metabolites were identified in Lijiang samples. Cross-model analysis of Diqing and Lijiang HAPH samples revealed 11 common differential metabolites between the two regions (Figure 4H). Correlation analysis of these 11 metabolites between the two regions revealed a level of association that differed between the regions (Figure 4I,J).

### 3.6. Differences in Metabolite Levels Between HAPH Patients and Healthy Controls

**Figure 5:**
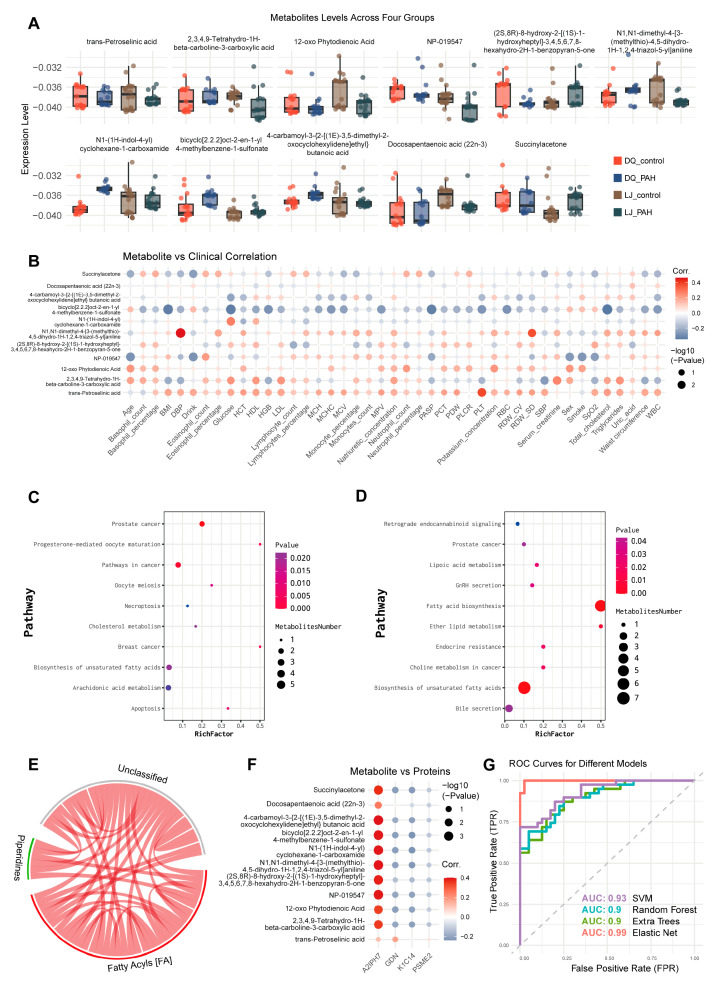
Metabolomic sequencing results revealed differences in disease and healthy groups between the two regions. (**A**) Expression of 11 metabolites in two groups of samples. (**B**) Bubble plot showed associations between 11 metabolites and clinical indicators of high-altitude pulmonary hypertension. (**C**,**D**) Kyoto Encyclopedia of Genes and Genomes (KEGG) showed differential metabolic enrichment pathways in two regions. (**E**) Chord diagram showed that the 11 metabolites are mainly distributed in three major classes such as fatty acyl groups. (**F**) Bubble plot showed associations between 4 proteins and 11 metabolites. (**G**) Multiple prediction models based on metabolomics.

We compared the metabolic level changes of 11 metabolites across the two groups and found that N1-cyclohexanecarboxamide and Bicyclo [2.2.2]oct-2-en-1-yl-4-methylbenzene-1-sulfonate were elevated in the disease groups from both regions, while docosapentaenoic acid and trans-petroselinic acid were decreased in the disease groups (Figure 5A). Correlation analysis between metabolites and clinical parameters revealed a significant positive correlation between dimethylaniline and pulmonary SP, and a positive correlation between succinylacetone and diastolic blood pressure (DBP), suggesting that these metabolites may serve as potential markers for diagnosing HAPH (Figure 5B). To explore the functions of these differential metabolites in the disease, we conducted Kyoto Encyclopedia of Genes and Genomes (KEGG) enrichment analysis, which showed that pathways such as unsaturated fatty acid synthesis were commonly enriched among the differential metabolites from both regions (Figure 5C,D). Metabolite component analysis indicated that the 11 metabolites were mainly distributed across three major categories, including acyl fatty acids (Figure 5E). Correlation analysis between the 11 metabolites and four proteins revealed a significant positive correlation between A2IPH7 and the 11 metabolites, while SERPINE2 showed a negative correlation with nine metabolites, except for succinylacetone, suggesting that these two proteins may be involved in or regulate the pathways associated with these nine metabolites (Figure 5F). Subsequently, we developed a predictive model for HAPH using various models, with the Elastic Net model achieving an area under the curve (AUC) of 0.99 (Figure 5G).

## 4. Discussion

This study compared the baseline characteristics of HAPH patients and control subjects from two regions, Diqing and Lijiang. The results showed no significant differences in age, sex, ethnicity, or most metabolic indicators between the HAPH and control groups in either region, indicating good comparability between the two populations.

Regarding cardiac structure, the right ventricular end-diastolic diameter (RVEDD) was significantly larger in the HAPH group than in the control group in both regions (Diqing: 21.5 ± 3.5 mm vs. 19.7 ± 1.5 mm, *p* = 0.049; Lijiang: 22.0 ± 3.1 mm vs. 20.0 ± 2.1 mm, *p* = 0.019). This finding is consistent with the pathophysiological features of HAPH: chronic hypoxia leads to pulmonary vascular remodeling and elevated pulmonary arterial pressure, resulting in compensatory right ventricular enlargement [19,20]. This suggests that right ventricular enlargement is a sensitive indicator of early cardiac structural changes in HAPH patients, highlighting the need for clinical monitoring of right heart function.

In terms of cardiac function, the left ventricular ejection fraction (LVEF) was significantly higher in the HAPH group than in the control group in Diqing (*p* = 0.004), but significantly lower in the HAPH group in Lijiang (*p* = 0.045). This discrepancy may be related to differences in hypoxic adaptation status due to varying altitudes, and further studies are needed to clarify the impact of HAPH on cardiac function.

Additionally, in the Lijiang region, systolic blood pressure was significantly elevated (*p* = 0.032) and hemoglobin levels were significantly decreased (*p* = 0.025) in the HAPH group, suggesting that blood pressure dysregulation and anemia may be involved in the disease process of HAPH, though the underlying mechanisms require further investigation.

In this study, we established a paired cohort consisting of 80 participants from 16 high-altitude regions in Yunnan, primarily composed of ethnic minorities such as the Naxi and Tibetan ethnic groups. Blood samples were collected, and multi-omics sequencing was performed using DIA label-free proteomics and LC-MS untargeted metabolomics. The proteomic results identified four proteins (A2IPH7, K1C14, PSME2, and SERPINE2) that showed significant differences in HAPH patients from both regions, which may serve as key candidate biomarkers for HAPH. SERPINE2 is significantly negatively correlated with TRV and pulmonary SP. This negative correlation was further validated in large cohort data from the UK Biobank and animal models. The metabolomic analysis identified 11 metabolites associated with the occurrence of HAPH, which were significantly correlated with the clinical data of the participants. The study found that differential metabolites between the disease and control groups were primarily enriched in unsaturated fatty acid synthesis pathways. Monitoring changes in the metabolic profile could aid in the early prediction of HAPH through simple methods. Finally, we developed machine learning models based on proteomic and metabolomic sequencing data, and multiple models demonstrated good predictive performance for HAPH occurrence. Our research contributes to the screening and prediction of HAPH and lays the foundation for further studies on its underlying mechanisms.

In recent years, proteomics has been widely applied to cardiovascular diseases, including heart failure, hypertension, and atherosclerosis [21,22,23,24,25]. Some studies have used omics to explore the changes in protein levels in PAH patients [14,15]. However, research and application of proteomics in HAPH are still lacking. In our study, we found that the SERPINE2 protein is negatively correlated with clinical indicators of HAPH. HAPH is characterized by increased pulmonary vascular resistance due to hypoxia-induced pulmonary vasoconstriction and pulmonary arteriolar remodeling [26,27]. These vascular changes involve all layers of the vessel wall, including endothelial dysfunction, smooth muscle extension into originally non-muscular vessels, and thickening of the adventitia [28,29]. Serine protease inhibitors play a key role in regulating cell adhesion, migration, proliferation, metabolism, and apoptosis due to their molecular flexibility and anti-protease activity [30]. As a serine protease inhibitor, SERPINE2 has traditionally been considered to play an important role in asthma and chronic obstructive pulmonary disease (COPD) [31,32]. Few studies have shown that this protein plays an important role in cardiovascular diseases [33]. However, recent evidence directly links SERPINE2 to pulmonary hypertension: genetic variants in SERPINE2 are associated with hypoxemia and elevated pulmonary artery pressure [34]. The SERPINE2 protein tightly binds to the extracellular matrix (ECM), and this binding is primarily mediated by heparan sulfate [35]. SERPINE2 bound to heparan sulfate inhibits the function of thrombin [36]. Studies have shown that thrombin is involved in regulating the response to vascular injury, and it has been demonstrated that thrombin can induce smooth muscle cells (SMCs) to synthesize collagen, which promotes ECM accumulation, as well as stimulate SMC contraction and proliferation [37,38]. Additionally, research has demonstrated that under hypoxic conditions, the NF-κB pathway contributes to the development of PAH. SERPINE2 modulates the expression of matrix metalloproteinases (MMPs) by inhibiting the NF-κB pathway, thereby promoting the degradation of the extracellular matrix (ECM) [39,40]. Of note, SIRT6 protects pulmonary microvascular endothelial cells from inflammation via NF-κB suppression [41], and SIRT3 activation ameliorates endothelial dysfunction by antagonizing mitochondrial damage [42]; these SIRT-mediated protective mechanisms parallel the anti-inflammatory action of SERPINE2, suggesting a potential synergistic role in maintaining pulmonary vascular homeostasis. Moreover, the crosstalk between mitochondrial dysfunction and fatty acid metabolism contributes to heart failure [43], and given that HAPH involves altered unsaturated fatty acid pathways, SERPINE2 may influence vascular remodeling through similar mitochondrial–metabolic axes. SERPINE2 may prevent pulmonary vascular constriction and proliferation by interacting with thrombin, but the mechanism by which SERPINE2 participates in the development of HAPH remains to be further investigated.

Metabolomics has also made significant progress in its application to cardiovascular diseases [13,44,45,46,47,48]. In this study, we identified that the differential metabolites between HAPH and normal samples are mainly involved in the unsaturated fatty acid synthesis pathway. Many clinical trials have confirmed that unsaturated fatty acids help reduce the incidence of cardiovascular diseases [49,50]; unsaturated fatty acids and their derivatives can regulate the synthesis and expression of various pro-inflammatory cytokines (such as TNF-α, IL-6, IL-8, IL-1β), which significantly exacerbate the inflammation and vascular remodeling in PAH [51]. Studies have shown that unsaturated fatty acids can reduce inflammation in the human pulmonary artery and dilate pulmonary blood vessels [52]. In addition, a clinical trial found that supplementation with unsaturated fatty acids can reduce the production of inflammatory factors in vivo [53]. In in vivo experiments, it was found that after exogenous supplementation with unsaturated fatty acids, the mean pulmonary artery pressure in rats decreased, vascular smooth muscle contraction and the extension of smooth muscle into originally non-muscular vessels were reduced, and right ventricular hypertrophy was less pronounced [54]. These findings suggest that unsaturated fatty acids may play a role in the prevention and treatment of HAPH. Mechanistically, the activation of the pro-inflammatory transcription factor NF-κB is reduced after supplementation with unsaturated fatty acids, leading to a decrease in the expression of pro-inflammatory genes (such as COX-2 and MCP-1). At the same time, the expression of matrix metalloproteinases (MMPs) is reduced. The lowered expression of these molecules helps to slow down the thickening and constriction of pulmonary vessels [55]. These findings further confirm that unsaturated fatty acids are involved in slowing the progression of HAPH. In this study, the levels of unsaturated fatty acid metabolism were found to be decreased in HAPH, and the role this plays in HAPH still needs further validation. The opposite changes in N1-cyclohexanecarboxamide between the two PH cohorts (increased in DQ but decreased in LJ) likely reflect cohort heterogeneity, including differences in disease etiology, medication history, genetic background, and sample size. This discrepancy highlights the need for larger, well-controlled validation studies before drawing firm conclusions about this metabolite as a universal PH biomarker. Although no direct evidence has yet established a regulatory relationship between SERPINE2 and unsaturated fatty acids, given that unsaturated fatty acids can influence key components of the fibrinolytic system (e.g., SERPINE1) and that SERPINE2 shares structural and functional similarities with SERPINE1, it is plausible that unsaturated fatty acids may modulate SERPINE2 expression and function through analogous transcriptional mechanisms [56].

It is estimated that 500.3 million people live in areas ≥1500 m above sea level, 81.6 million people live in areas ≥2500 m and almost all of these areas belong to low- and middle-income countries [57]. Given that HAPH is currently significantly understudied globally, and to address the rising prevention, treatment, and management challenges of HAPH [10], our findings provide promising perspectives for further clarifying the development of HAPH and targeted interventions.

The study has the following advantages: All participants in this study were from the Yunnan Plateau region. The research investigates plasma samples from this unique population, which are difficult to obtain. The study covered a wide range of sampling areas and regions, with minimal systematic bias. Additionally, the samples were paired strictly for sequencing, ensuring the reliability of the sequencing data. Moreover, 90% of our blood samples come from ethnic minority groups, including the Naxi and Tibetan populations, providing significant omics data support for multi-ethnic studies. However, there are some major limitations to the study: Firstly, the relatively small sample size may limit the generalizability of our findings. Secondly, due to logistic constraints in this high-altitude community-based study, screening of PAH is based on echocardiography through Doppler-derived TRV and other echocardiographic markers, which is not the gold standard for diagnosing pulmonary hypertension. Thirdly, the follow-up data available for this study are limited, so its predictive ability regarding long-term patient prognosis is insufficient. Lastly, the exploration and validation of mechanisms are inadequate. Specifically, although we confirmed the altered expression of SERPINE2 in the rat HAPH model by immunofluorescence, the direct functional link between SERPINE2 and the identified metabolites (e.g., unsaturated fatty acids, N1-cyclohexanecarboxamide) remains unclear. Future studies involving in vitro experiments (e.g., SERPINE2 knockdown or overexpression in pulmonary arterial cells) combined with targeted metabolomics are needed to provide translational medical evidence for HAPH intervention. Additionally, the modest sample size (40 HAPH patients and 40 controls) and the lack of false discovery rate (FDR) correction for multiple testing in our high-dimensional omics analyses represent additional limitations, as they may increase the risk of false-positive findings; independent validation in larger cohorts is therefore necessary. Finally, it should be noted that the pathophysiological mechanisms of high-altitude pulmonary hypertension (HAPH) and idiopathic pulmonary arterial hypertension (IPAH) are distinct; therefore, using IPAH data from the UK Biobank to validate our HAPH findings is another limitation. Direct validation in independent HAPH cohorts is required.

## 5. Conclusions

In conclusion, we established a rigorously matched cohort and identified significant proteins and metabolic factors interacting during the development of HAPH. This study highlights the dual role of SERPINE2 and unsaturated fatty acid metabolites in potentially alleviating the severity of the disease. We also developed machine learning models using sequencing data to predict the occurrence of HAPH. By linking protein molecules and metabolic components, this study offers a more comprehensive perspective on the potential for HAPH treatment and personalized medical strategies.

## Figures and Tables

**Table 1:** Characteristics of HAPH and control groups in high altitude areas.

		Diqing (*n* = 36)			Lijiang (*n* = 44)		
		HAPH (*n* = 18)	Control (*n* = 18)	*p*-Value	HAPH (*n* = 22)	Control (*n* = 22)	*p*-Value
Age, yr		64.3 ± 7.9	65.4 ± 10.0	0.701	55.0 ± 10.4	55.3 ± 10.4	0.942
Sex	Male	5 (27.8)	4 (22.2)	0.700	2 (9.1)	2 (9.1)	1.000
	Female	13 (72.2)	14 (77.8)		20 (90.9)	20 (90.9)	
Ethnic group	Han Chinese	2 (11.1)	3 (16.7)	0.525	2 (9.1)	1 (4.5)	0.599
	Tibetan	14 (77.8)	15 (83.3)		0 (0.0)	0 (0.0)	
	Naxi	1 (5.6)	0 (0.0)		14 (63.6)	17 (77.3)	
	Other Ethnic minorities	1 (5.6)	0 (0.0)		6 (27.3)	4 (18.2)	
Duration of residence, yr	54.4 ± 17.7	61.0 ± 16.3	0.252	0.252	48.8 ± 18.2	49.5 ± 15.6	0.895
Smoker		4 (22.2)	3 (16.7)	1.000	1 (4.5)	0 (0.0)	1.000
Drinker ^#^		2 (11.1)	2 (11.1)	1.000	0 (0.0)	0 (0.0)	/
Waist measurement, cm		82.9 ± 8.7	84.7 ± 9.3	0.551	83.4 ± 8.3	81.2 ± 7.3	0.359
BMI, kg/m^2^		22.5 ± 3.0	23.3 ± 3.0	0.435	23.3 ± 4.4	22.4 ± 3.1	0.470
SBP, mmHg		127.4 ± 8.8	126.3 ± 18.0	0.815	129.8 ± 11.0	121.8 ± 12.9	0.032 *
DBP, mmHg		73.2 ± 7.5	75.3 ± 9.1	0.458	74.7 ± 8.5	73.0 ± 10.4	0.554
TC, mmol/L		5.0 ± 1.0	5.0 ± 0.7	0.824	5.4 ± 0.9	5.2 ± 1.1	0.513
TG, mmol/L		1.2 ± 0.8	1.3 ± 0.5	0.538	1.3 ± 1.2	1.2 ± 0.5	0.774
HDL-C, mmol/L		1.7 ± 0.4	1.5 ± 0.2	0.226	1.9 ± 0.5	1.7 ± 0.4	0.052
FBG, mmol/L		5.1 ± 0.9	5.6 ± 2.2	0.384	5.4 ± 0.8	5.7 ± 2.4	0.532
Hemoglobin, g/L		155.3 ± 22.6	148.7 ± 22.0	0.380	152.0 ± 21.1	170.3 ± 30.4	0.025 *
Erythrocyte, ×10^12^/L		4.9 ± 0.8	5.0 ± 0.6	0.966	5.3 ± 1.0	5.7 ± 1.0	0.178
SCr, μmol/L		69.7 ± 11.9	66.1 ± 13.5	0.409	73.7 ± 21.4	71.9 ± 22.5	0.785
UA, μmol/L		351.2 ± 99.9	335.7 ± 72.0	0.597	332.2 ± 96.1	344.6 ± 74.3	0.633
Oxygen saturation <90%		9 (50.0)	7 (38.9)	0.502	5 (22.7)	2 (9.1)	0.412
Atrial fibrillation ^#^		0	0	/	1 (4.5)	0 (0.0)	1.000
Left atrial internal diameter, mm		34.7 ± 4.6	33.0 ± 2.7	0.194	33.8 ± 4.4	31.8 ± 6.6	0.247
Right ventricular end-diastolic diameter, mm		21.5 ± 3.5	19.7 ± 1.5	0.049 *	22.0 ± 3.1	20.0 ± 2.1	0.019 *
Left ventricular end-diastolic diameter, mm		45.9 ± 2.7	45.9 ± 5.3	1.000	44.6 ± 7.4	44.8 ± 4.4	0.903
Left ventricular ejection (%)		63.9 ± 4.0	59.6 ± 4.4	0.004 **	63.55 ± 4.1	65.9 ± 3.5	0.045 *

The data in the table are expressed as mean and standard deviation for continuous variables, numbers (percentages) for categorical variables. HAPH: High-altitude pulmonary hypertension. ^#^ The number of events was 0 for both groups, so the statistics and statistical significance could not be reported. * *p* < 0.05; ** *p* < 0.01.

## Data Availability

The raw data supporting the conclusions of this article will be made available by the authors on request. The datasets generated or analyzed during the current study are available at: https://docs.google.com/spreadsheets/d/1AeeADtT0U1AukliiNyiVzVRdLYPkTbruQSk38DeutU8/edit?gid=1450719288#gid=1450719288 https://ftp.ebi.ac.uk/pub/databases/gwas/summary_statistics/GCST90241001-GCST90242000/GCST90241257/harmonised/GCST90241257.h.tsv.gz (accessed on 6 June 2024). The download links for the GWAS summary data used in this study are as follows: pulmonary arterial hypertension: https://ftp.ebi.ac.uk/pub/databases/gwas/summary_statistics/GCST007001-GCST008000/GCST007228/ (accessed on 6 June 2024). eQTL summary data of GDN: https://console.cloud.google.com/storage/browser/gtex-resources/GTEx_Analysis_v8_QTLs/GTEx_Analysis_v8_EUR_eQTL_all_associations?pageState=(%22StorageObjectListTable%22:(%22f%22:%22%255B%257B_22k_22_3A_22_22_2C_22t_22_3A10_2C_22v_22_3A_22_5C_22whole_5C_22_22%257D%255D%22))&inv=1&invt=AbzUJw&prefix=&forceOnObjectsSortingFiltering=true; (accessed on 6 June 2024). pQTL summary data of GDN: https://ftp.ebi.ac.uk/pub/databases/gwas/summary_statistics/GCST90241001-GCST90242000/GCST90241257/harmonised/ (accessed on 6 June 2024). The datasets generated and/or analyzed during the current study are not publicly available due to patient privacy and confidentiality protection, but are available from the corresponding author on reasonable request.

## Supplementary Materials

The following supporting information can be downloaded at: https://www.mdpi.com/article/10.3390/jcdd13050195/s1.

## Abbreviations

SBP	Systolic blood pressure
DBP	Diastolic blood pressure
TC	Total Cholesterol
TG	Triglycerides
LDL-C	Low-density lipoprotein
HDL-C	High-density lipoprotein
FBG	Fasting blood glucose
SCr	Serum creatinine
UA	Uric acid
b	beta coefficient
nsnp	number of single nucleotide polymorphisms (SNPs)
se	standard error
