# Supplementary material for: Multi-Omics Identification of Biomarkers for High-Altitude Pulmonary Hypertension
Source: J Cardiovasc Dev Dis. 2026 Apr 30;13(5):195. doi: 10.3390/jcdd13050195 (PMC13207707; doi:10.3390/jcdd13050195)
Supplement: Supplementary file 1 [file jcdd-13-00195-s001.zip › jcdd-4147863-supplementary.pdf]

Multi-omics Identification of Biomarkers for High-altitude Pulmonary Hypertension.

Zhe Chen[1]†, Linhong Pang[2, 3]†, Yidan Zheng[1], Li Xu[1], Mingjing Tang[2], Ziwen Zhao[2], Tianyu Wang[4], Jin Li[4], Yunfei Zhou[4], Lin Duo[2], Wenlong Zhu[2], Zhiling Luo[4], Fei Li[1,2#], Da Zhu[2#].

[1] Department of Cardiovascular Surgery, Union Hospital, Tongji Medical College, Huazhong University of Science and Technology, Wuhan, China.

[2]Department of Structural Heart Center, Fuwai Yunnan Cardiovascular Hospital, Chinese Academy of Medical Sciences, Affiliated Cardiovascular Hospital of Kunming Medical University, Kunming, China.

[3] School of Health Policy and Management, Chinese Academy of Medical Sciences & Peking Union Medical College, Beijing, China.

[4] Department of Echocardiogram, Fuwai Yunnan Hospital, Chinese Academy of Medical Sciences, Affiliated Cardiovascular Hospital of Kunming Medical University, Kunming, China

† These authors contributed equally to this work

[#] Dr. Zhu and Dr Li are joint corresponding authors.

[#] Corresponding authors:

Da Zhu, [2]Department of Structural Heart Disease, Yunnan Fuwai

Cardiovascular Hospital, Kunming Medical University, 528 Shahebei Rd, 65000 Kunming, China. Email: zhuda8687@126.com

Fei Li, [1]Department of cardiovascular surgery, Union Hospital, Tongji Medical College, Huazhong University of Science and Technology, 1277 Jiefang Ave, Wuhan 430022, China. [2]Department of Cardiac Surgery, Yunnan Fuwai Cardiovascular Hospital, Kunming Medical University, 528 Shahebei Rd, 65000 Kunming, China. Email: lifei_union@hust.edu.cn

The category of the manuscript: Original Article

This file includes:

Supplementary Figures S1-S5

Supplementary Tables

1. Supplementary Figures

1.1 Supplementary Figure S1

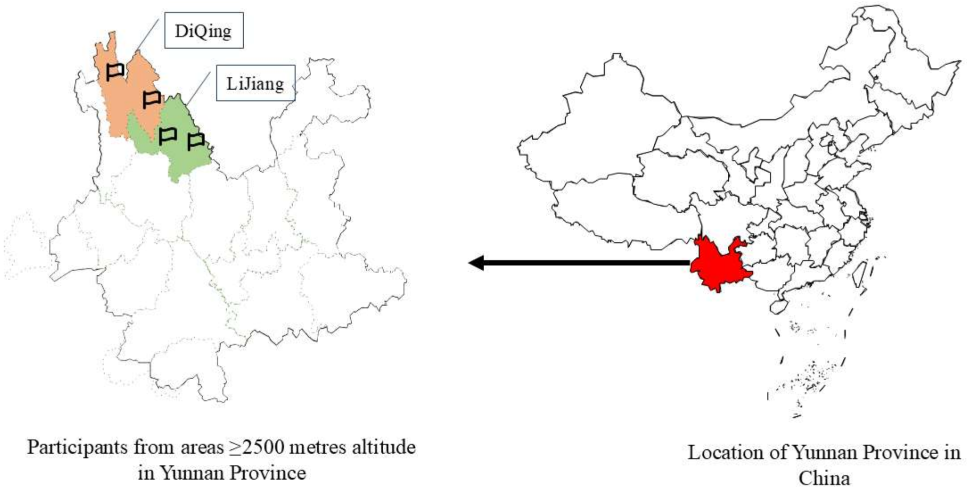

Figure S1 The survey locations of Diqing Tibetan Autonomous Prefecture and Lijiang City.

1.2 Supplementary Figure S2

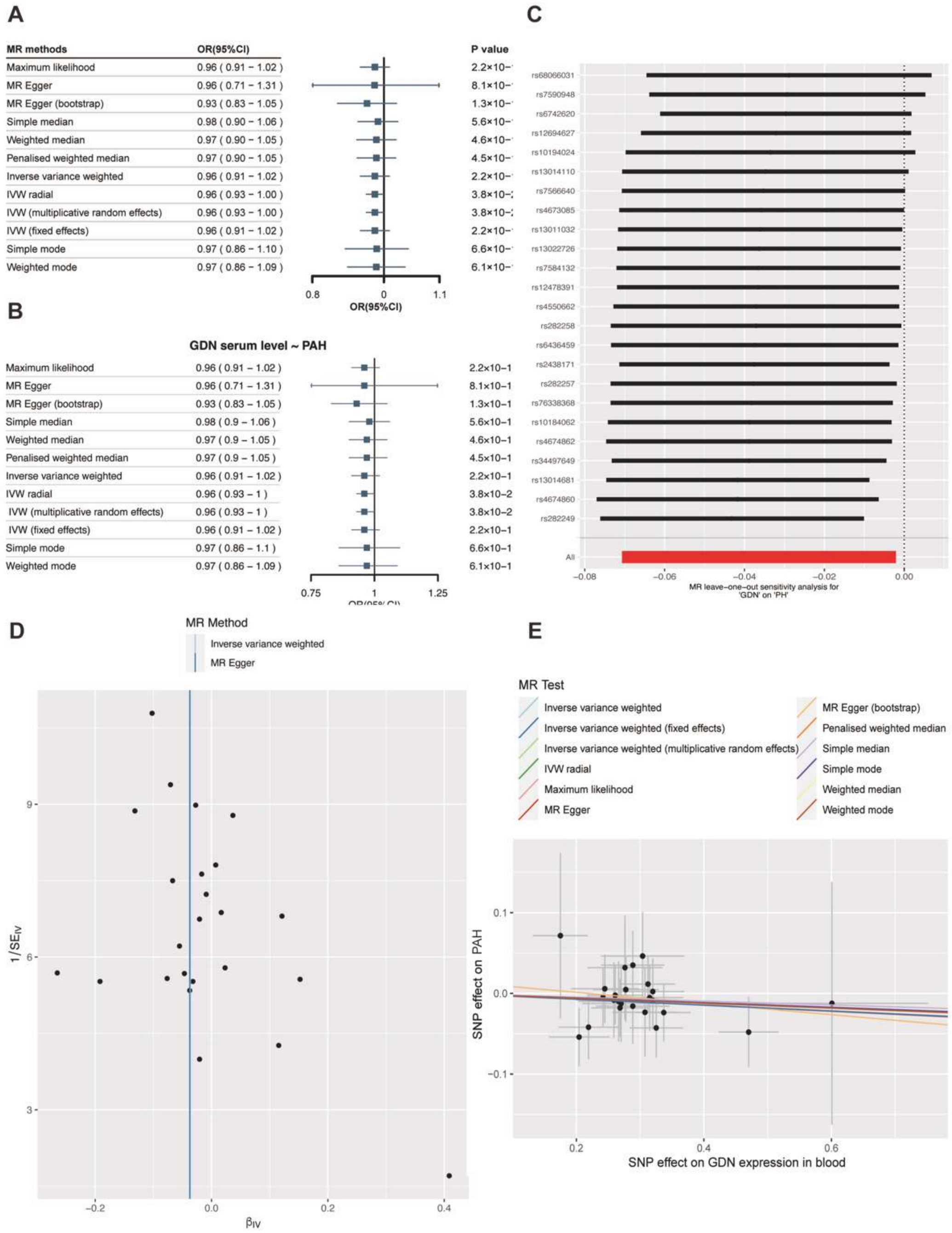

Figure S2 eQTL results predicted by multiple MR models. (A-B) The forest plots for the eQTL analyses were generated after applying a logarithmic transformation to the axes. (C) Use the leave-one-out method to vertically plot the eQTL MR results. (D-E) eQTL funnel plot and scatter plot results.

1.3 Supplementary Figure S3

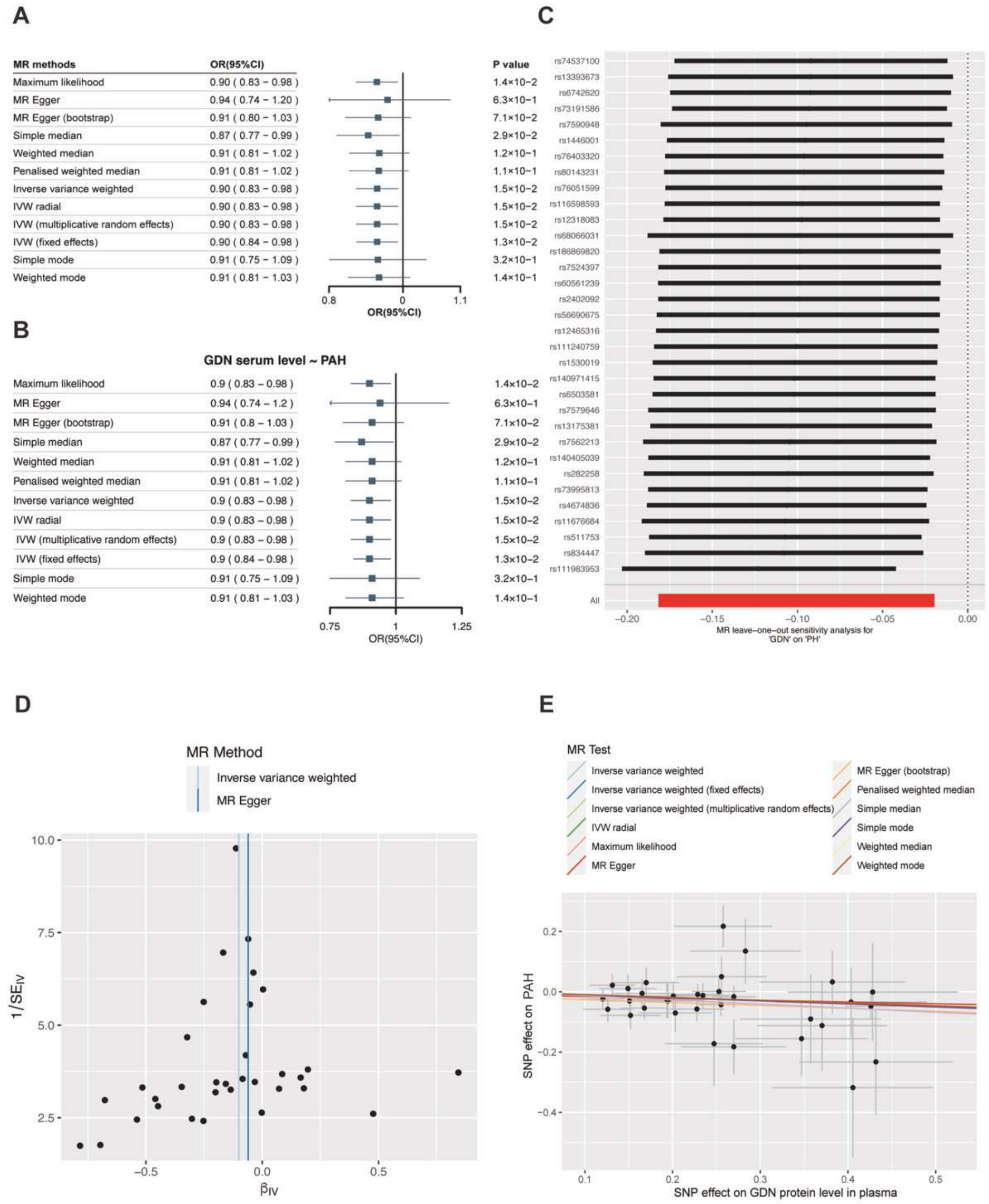

Figure S3 pQTL results predicted by multiple MR models. (A-B) The forest plots for the pQTL analyses were generated after applying a logarithmic transformation to the axes. (C) Use the leave-one-out method to vertically plot the pQTL MR results. (D-E) pQTL funnel plot and scatter plot results.

1.4 Supplementary Figure S4

STROBE Statement—Checklist of items that should be included in reports of ***cohort studies***

Item No **Recommendation** **Paragraph**

Title and abstract 1 (*a*) Indicate the study's design with a commonly used term in the title or the abstract Title and Abstract

(*b*) Provide in the abstract an informative and balanced summary of what was done and what was found Abstract

Introduction

Background/rationale 2 Explain the scientific background and rationale for the investigation being reported Paragraph 1, 2 and 3

Objectives 3 State specific objectives, including any prespecified hypotheses Paragraph 4

Methods

Study design 4 Present key elements of study design early in the paper Figure 1

Setting 5 Describe the setting, locations, and relevant dates, including periods of recruitment, exposure, follow-up, and data collection 3.1 Epidemiological survey

Participants 6 (*a*) Give the eligibility criteria, and the sources and methods of selection of participants. Describe methods of follow-up 3.1 Epidemiological survey

(*b*) For matched studies, give matching criteria and number of exposed and unexposed

Variables 7 Clearly define all outcomes, exposures, predictors, potential confounders, and effect modifiers. Give diagnostic criteria, if applicable 3.1 Epidemiological survey;
3.4 Genome-wide association study data;
3.6 Mendelian randomization analysis and sensitivity analysis;
3.10 Proteomics analysis

Data sources/ measurement 8* For each variable of interest, give sources of data and details of methods of assessment (measurement). Describe comparability of assessment methods if there is more than one group 3.1 Epidemiological survey;
3.4 Genome-wide association study data;
3.10 Proteomics analysis;
3.11 DIA Protein Quantification and Statistical Analysis;
3.12 Differential Expression Clustering

Bias 9 Describe any efforts to address potential sources of bias 3.1 Epidemiological survey;
3.2 Quality control of Genotype and imputation data;
3.6 Mendelian randomization analysis and sensitivity analysis;
3.7 Animal experiments;
3.8 Immunofluorescence staining

Study size 10 Explain how the study size was arrived at 3.1 Epidemiological survey;
3.5 Data source of MR analysis;
3.6 Mendelian randomization analysis and sensitivity analysis

Quantitative variables 11 Explain how quantitative variables were handled in the analyses. If applicable, describe which groupings were chosen and why 3.10 Proteomics analysis;
3.11 DIA Protein Quantification and Statistical Analysis;
3.12 Differential Expression Clustering

Statistical methods 12 (*a*) Describe all statistical methods, including those used to control for confounding
3.6 Mendelian randomization analysis and sensitivity analysis;
3.11 DIA Protein Quantification and Statistical Analysis

(*b*) Describe any methods used to examine subgroups and interactions 3.12 Differential Expression Clustering

(*c*) Explain how missing data were addressed 3.1 Epidemiological survey;
3.2 Quality control of Genotype and imputation data;
3.4 Genome-wide association study data;
3.5 Data source of MR analysis;
3.10 Proteomics analysis

Figure S4 STROBE Statement—Checklist of items that should be included in reports of *cohort studies*

1.5 Supplementary Figure S5

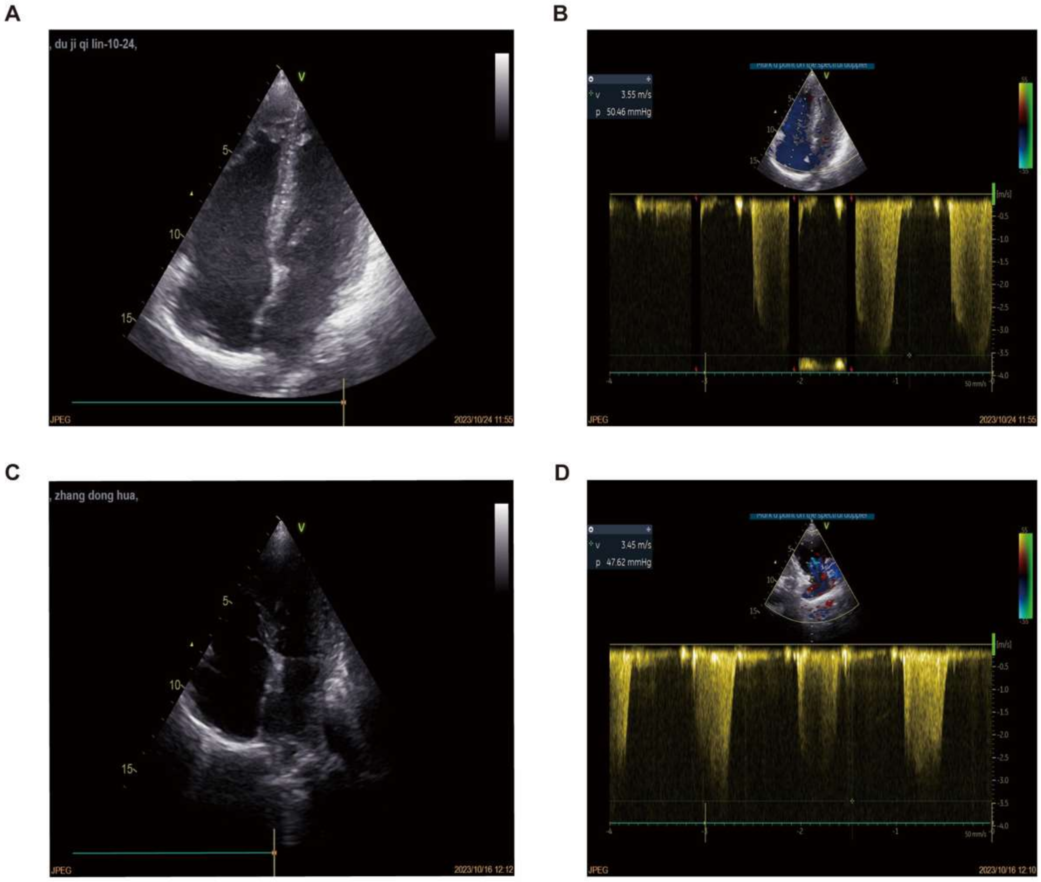

Figure S5 Representative echocardiographic images from two HAPH patients in Lijiang. (A-D) The images demonstrate characteristic findings of high-altitude pulmonary hypertension, including increased right ventricular (RV) dimensions, abnormal septal motion, and tricuspid regurgitation.

2. Supplementary Tables

2.1 Supplementary Tables S1-S2

Supplementary Table S1 The Pleiotropy of eQTL

id.exposure	id.outcome	outcome	exposure	egger_intercept	se	pval	MR_PRESSO_p
SERPINE2	PAH	PAH	SERPINE2	0.00035011	0.04581796	0.99397205	1

Supplementary Table S2 The heterogeneity of eQTL

id.exposure	id.outcome	outcome	exposure	method	Q	Q_df	Q_pval
SERPINE2	PAH	PAH	SERPINE2	MR Egger	7.97399116	22	0.99722839
SERPINE2	PAH	PAH	SERPINE2	Inverse variance weighted	7.97404955	23	0.99841363

2.2 Supplementary Tables S3-S4

Supplementary Table S3 The Pleiotropy of pQTL

id.exposure	id.outcome	outcome	exposure	egger_intercept	se	pval	MR_PRESSO_p
SERPINE2	PAH	PAH	SERPINE2	-0.0095973	0.02795757	0.7337037	0.431

Supplementary Table S4 The heterogeneity of pQTL

id.exposure	id.outcome	outcome	exposure	method	Q	Q_df	Q_pval
SERPINE2	PAH	PAH	SERPINE2	MR Egger	33.1175599	31	0.36415839
SERPINE2	PAH	PAH	SERPINE2	Inverse variance weighted	33.2434519	32	0.40646306

2.3 Supplementary Tables S5-S6 The SNP loci table of eQTL and pQTL

SNP	effect_allele.exposure	other_allele.exposure	effect_allele.outcome	other_allele.outcome	beta.exposure	beta.outcome	eaf.exposure	eaf.outcome	outcome	pval.outcome	exposure	pval.exposure
rs10184062	C	G	C	G	-0.27755854	-0.004626282	0.24910396	0.256817	PAH	0.911975	SERPINE2	1.111631167386 72e-07
rs10194024	A	C	A	C	-0.33	0.0237868366455082	0.4327957	0.555666	PAH	0.512689	SERPI	1.304086138380 41e-14

					7055<br>1						NE<br>2	
rs12<br>478<br>391	A	C	A	C	-<br>0.60<br>0703<br>84	0.01238<br>7951499<br>8389	0.01<br>9713<br>262	0.02<br>133<br>7	PA<br>H	0.94<br>3743	SE<br>RPI<br>NE<br>2	7.70885<br>2382924<br>22e-05
rs12<br>694<br>627	T	C	T	C	-<br>0.21<br>9017<br>8	0.04198<br>5157783<br>5069	0.30<br>0179<br>18	0.70<br>122<br>4	PA<br>H	0.28<br>8223	SE<br>RPI<br>NE<br>2	3.95448<br>0618217<br>51e-06

rs13011032	A	C	A	C	0.2701743	-0.012620302	0.15143369	0.154823	PAH	0.803162	SERPINE2	1.14675502091107e-05
rs13014110	T	G	T	G	0.26848894	-0.017970507	0.33691755	0.34543	PAH	0.634661	SERPINE2	5.45425182363581e-09
rs13014681	A	C	A	C	0.3038511	0.0462345203395346	0.123655915	0.124599	PAH	0.400744	SERPI	4.38619722044804e-06

											NE2	
rs13022726	C	T	C	T	0.26619908	-0.009980641	0.13530466	0.140038	PAH	0.850539	SERPINE2	2.51866575909333e-05
rs2438171	G	T	G	T	-0.17496286	-0.071496002	0.4722222	0.4775	PAH	0.565052	SERPINE2	5.88088278239532e-05

rs282249	A	G	A	G	0.28856653	0.0349413775360061	0.23297492	0.232886	PAH	0.413144	SERPINE2	1.17280987853406e-08
rs282257	A	G	A	G	-0.2609112	0.0023871484924981	0.35573477	0.635127	PAH	0.948998	SERPINE2	6.34200559539535e-09
rs282258	C	T	C	T	-0.31	0.00870797529056603	0.42114693	0.575949	PAH	0.811809	SERPI	3.56393110144171e-13

					98218						NE2	
rs34497649	A	G	A	G	0.27618673	0.0318920005463249	0.15143369	0.153351	PAH	0.633798	SERPINE2	3.65044627889147e-06
rs4550662	C	G	C	G	0.24172895	-0.004998472	0.36290324	0.374048	PAH	0.893527	SERPINE2	9.01421275141302e-08

rs4673085	A	G	A	G	0.28848195	-0.015894653	0.160394027	0.164808	PAH	0.748036	SERPINE2	9.05772377147351e-07
rs4674860	T	C	T	C	0.31225944	0.0114571158743147	0.4408602	0.447713	PAH	0.753096	SERPINE2	8.5940441616465e-13
rs4674862	T	A	T	A	0.31984168	0.00229237050985138	0.24283154	0.239474	PAH	0.95706	SERPI	1.83335778746642e-10

											NE2	
rs6436459	T	C	T	C	0.31489918	-0.00534526	0.22759856	0.23203	PAH	0.901682	SERPINE2	4.321178021918 22e-09
rs6742620	C	T	C	T	0.20422985	-0.054152114	0.3091398	0.309783	PAH	0.16917	SERPINE2	2.004744712802 26e-05

rs68066031	C	T	C	T	-0.47016987	0.0480413124213673	0.2392473	0.233415	PAH	0.26749	SERPINE2	1.2799367271608e-21
rs7566640	T	C	T	C	-0.30758825	0.0234674721683312	0.24193549	0.755494	PAH	0.680097	SERPINE2	3.27740717327031e-09
rs7584132	A	G	A	G	-0.25	0.00831433988407679	0.16397852	0.832631	PAH	0.864565	SERPI	1.46840282381077e-05

					8830<br>34						NE<br>2	
rs75<br>909<br>48	A	G	A	G	-<br>0.32<br>5224<br>28	0.04284<br>2990930<br>546	0.44<br>8924<br>72	0.54<br>292<br>3	PA<br>H	0.23<br>9133	SE<br>RPI<br>NE<br>2	6.43420<br>2216569<br>23e-14
rs76<br>338<br>368	A	G	A	G	-<br>0.24<br>4610<br>43	-<br>0.00568<br>9153	0.20<br>6989<br>24	0.21<br>225<br>5	PA<br>H	0.89<br>7984	SE<br>RPI<br>NE<br>2	5.06542<br>2668651<br>79e-06

SNP	effect_allele.exposure	other_allele.exposure	effect_allele.outcome	other_allele.outcome	beta.exposure	beta.outcome	eaf.exposure	eaf.outcome	outcome	pval.outcome	exposure	pval.exposure
rs111240759	T	C	T	C	-0.4035	0.034104764	NA	0.02361	PAH	0.782817	SERPINE2	2.88E-06
rs111983953	A	G	A	G	-0.2577	-0.217324266	NA	0.046322	PAH	0.021176	SERPINE2	4.07E-06
rs116598593	T	C	T	C	0.4056	-0.317633759	NA	0.985479	PAH	0.122367	SERPINE2	9.33E-06
rs11676684	T	C	T	C	-0.2528	-0.000931434	NA	0.213188	PAH	0.983214	SERPINE2	2.29E-16

rs12318083	A	C	A	C	0.2473	-0.172100331	NA	0.960852	PAH	0.199928	SERPINE2	8.71E-06
rs12465316	T	G	T	G	0.1934	-0.026188957	NA	0.917209	PAH	0.68659	SERPINE2	8.13E-06
rs13175381	A	G	A	G	0.1491	0.01079809	NA	0.830762	PAH	0.822318	SERPINE2	5.13E-06
rs13393673	A	G	A	G	-0.2277	0.057294423	NA	0.791592	PAH	0.200401	SERPINE2	2.09E-13
rs140405039	T	C	T	C	-0.3822	-0.032768056	NA	0.024127	PAH	0.785605	SERPINE2	4.07E-06

rs140971415	T	C	T	C	0.4281	-0.000858368	NA	0.018321	PAH	0.996494	SERPINE2	1.00E-05
rs1446001	T	C	T	C	0.1261	-0.057932059	NA	0.743952	PAH	0.159529	SERPINE2	8.51E-06
rs1530019	T	C	T	C	0.2008	-0.014131676	NA	0.160977	PAH	0.775744	SERPINE2	4.79E-09
rs186869820	A	G	A	G	-0.3704	0.111960786	NA	0.029633	PAH	0.471241	SERPINE2	5.13E-07
rs2402092	T	C	T	C	-0.3574	0.09028576	NA	0.027552	PAH	0.565524	SERPINE2	8.13E-06

rs282258	T	C	T	C	0.2286	-0.008707975	NA	0.424051	PAH	0.811809	SERPINE2	6.31E-20
rs4674836	A	G	A	G	0.1313	0.021700825	NA	0.616871	PAH	0.559182	SERPINE2	2.69E-07
rs511753	A	G	A	G	-0.2831	-0.135037757	NA	0.035324	PAH	0.198147	SERPINE2	6.92E-06
rs56690675	A	G	A	G	0.1947	-0.030497353	NA	0.09433	PAH	0.625289	SERPINE2	1.62E-06
rs60561239	A	G	A	G	-0.1508	0.030289603	NA	0.169452	PAH	0.528662	SERPINE2	6.76E-06

rs6503581	A	G	A	G	0.1652	-0.005251188	NA	0.846474	PAH	0.915915	SERPINE2	1.35E-06
rs6742620	T	C	T	C	-0.1678	0.054152114	NA	0.690217	PAH	0.16917	SERPINE2	6.92E-10
rs68066031	T	C	T	C	0.4261	-0.048041312	NA	0.766585	PAH	0.26749	SERPINE2	1.07E-46
rs73191586	A	G	A	G	0.152	-0.078282674	NA	0.14498	PAH	0.133182	SERPINE2	1.00E-05

rs73995813	A	C	A	C	-0.1699	-0.030431373	NA	0.123913	PAH	0.587771	SERPINE2	5.75E-06
rs74537100	A	G	A	G	0.2698	-0.182492376	NA	0.953866	PAH	0.025659	SERPINE2	6.03E-06
rs7524397	T	C	T	C	0.1204	-0.023751855	NA	0.40321	PAH	0.520474	SERPINE2	1.74E-06
rs7562213	A	G	A	G	0.2698	-0.016225649	NA	0.352201	PAH	0.665908	SERPINE2	7.24E-27

rs7579646	A	G	A	G	0.2345	-0.01234691	NA	0.203746	PAH	0.781872	SERPINE2	2.45E-14
rs7590948	A	G	A	G	-0.2552	0.042842991	NA	0.542923	PAH	0.239133	SERPINE2	2.95E-24
rs76051599	T	C	T	C	-0.4318	0.232523422	NA	0.021606	PAH	0.154116	SERPINE2	8.32E-07
rs76403320	A	G	A	G	0.347	-0.155391338	NA	0.977559	PAH	0.18926	SERPINE2	3.98E-06
rs80143231	A	C	A	C	0.2031	-0.070322387	NA	0.903626	PAH	0.242818	SERPINE2	1.45E-06

rs834447	A	G	A	G	-0.2558	-0.050213912	NA	0.928607	PAH	0.462552	SERPINE2	5.62E-07

2.4 Supplementary Tables S7-S8 The forest plot data for eQTL and pQTL

id.exposure	id.outcome	outcome	exposure	method	nsnp	b	se	pval

SERPINE2	PAH	PAH	SERPINE2	Maximum likelihood	24	-0.036292055	0.0298769326107244	0.224473574183512
SERPINE2	PAH	PAH	SERPINE2	MR Egger	24	-0.037545167	0.155679477030059	0.811657125021914
SERPINE2	PAH	PAH	SERPINE2	MR Egger (bootstrap)	24	-0.067940599	0.060723040190334	0.133
SERPINE2	PAH	PAH	SERPINE2	Simple median	24	-0.023952793	0.040842178993062	0.557558390689509

SERPINE2	PAH	PAH	SERPINE2	Weighted median	24	-0.030175311	0.0404728051562838	0.455927189442111
SERPINE2	PAH	PAH	SERPINE2	Penalised weighted median	24	-0.030175311	0.0395527124354005	0.44551479601894
SERPINE2	PAH	PAH	SERPINE2	Inverse variance weighted	24	-0.036377435	0.029708877513403	0.220777442638232
SERPINE2	PAH	PAH	SERPINE2	IVW radial	24	-0.036375754	0.0174938265842007	0.0375853710793398

SERPINE2	PAH	PAH	SERPINE 2	Inverse variance weighted (multiplicative random effects)	24	-0.036377435	0.0174928990642475	0.0375664246721029
SERPINE2	PAH	PAH	SERPINE 2	Inverse variance weighted (fixed effects)	24	-0.036377435	0.029708877513403	0.220777442638232
SERPINE2	PAH	PAH	SERPINE 2	Simple mode	24	-0.028831456	0.0647281118547665	0.660177660573903

SERPINE2	PAH	PAH	SERPINE2	Weighted mode	24	-0.030521754	0.0589107191754119	0.60933557863486
id.exposure	id.outcome	outcome	exposure	method	nsnp	b	se	pval
SERPINE2	PAH	PAH	SERPINE2	Maximum likelihood	33	-0.101660063	0.0413019814916804	0.0138401898719076
SERPINE2	PAH	PAH	SERPINE2	MR Egger	33	-0.060306014	0.124571716523194	0.631712683272368

SERPINE2	PAH	PAH	SERPINE2	MR Egger (bootstrap)	33	-0.096722758	0.0646917523763194	0.071
SERPINE2	PAH	PAH	SERPINE2	Simple median	33	-0.135413426	0.0621819213833211	0.029428551886169
SERPINE2	PAH	PAH	SERPINE2	Weighted median	33	-0.092002211	0.0592205384948735	0.120291217458656
SERPINE2	PAH	PAH	SERPINE2	Penalised weighted median	33	-0.094281236	0.0588525279122501	0.109157538208289

SERPINE2	PAH	PAH	SERPINE 2	Inverse variance weighted	33	- 0.10057409 4	0.041347189330710 3	0.014997940002212 9
SERPINE2	PAH	PAH	SERPINE 2	IVW radial	33	- 0.10064982 5	0.041344030312030 9	0.014914588866230 3
SERPINE2	PAH	PAH	SERPINE 2	Inverse variance weighted (multiplicativ e random effects)	33	- 0.10057409 4	0.041347189330710 3	0.014997940002212 9

SERPINE2	PAH	PAH	SERPINE2	Inverse variance weighted (fixed effects)	33	-0.100574094	0.0405665360708439	0.0131663453300432
SERPINE2	PAH	PAH	SERPINE2	Simple mode	33	-0.097634505	0.0959586001342932	0.316563734757369
SERPINE2	PAH	PAH	SERPINE2	Weighted mode	33	-0.093152557	0.0610922394225894	0.137136040445724

Statistical methods

All statistical analyses were performed using R software (version 4.3.1). The main R packages used in this study are listed below. A complete list of all loaded packages and their versions is provided at the end of this document.

Differential expression analysis (proteomics and metabolomics):

The limma package (version 3.56.2) was used to fit linear models and compute moderated t-statistics for each protein or metabolite. Differentially expressed features were identified based on **absolute $\log_2$ fold change ≥ 1** and **raw P value < 0.05**. No adjustment for multiple comparisons (e.g., FDR) was applied.

Pathway enrichment analysis:

Over-representation analysis was performed using clusterProfiler (version 4.8.3) and DOSE (version 3.26.1). Pathways with **raw P value < 0.05** were considered enriched. The fgsea package (version 1.27.1) was used for gene set enrichment analysis with the same significance threshold.

Machine learning models:

Random Forest and Elastic Net models were built using randomForest (version 4.7-1.1) and glmnet (version 4.1-4). Model performance was evaluated using **10-fold cross-validation** (implemented with foreach version 1.5.2 and doParallel version 1.0.17). The area under the ROC

curve (AUC) was calculated for each fold, and the mean AUC across folds was reported. No multiple testing correction was applied to the AUC values.

Correlation and group comparisons:

Pearson or Spearman correlation tests were performed using rstatix (version 0.7.2). For two-group comparisons, Student's t-test or Mann-Whitney U test was used. For multi-group comparisons, one-way ANOVA followed by Tukey's post-hoc test was applied (ggpubr version 0.6.0, car version 3.1-2). A **raw P value < 0.05** was considered statistically significant in all cases.

Visualization:

Figures were generated using ggplot2 (version 3.4.3), ComplexHeatmap (version 2.16.0), ggrepel (version 0.9.3), ggpubr, and scales (version 1.3.0).

Full package list:

The following packages were loaded during the analysis (listed by namespace, including dependencies). Version numbers are indicated where available.

[1] RColorBrewer_1.1-3 shape_1.4.6 rstudioapi_0.15.0

[4] jsonlite_1.8.7	umap_0.2.10.0	MultiAssayExperiment_1.26.0
[7] magrittr_2.0.3	farver_2.1.1	GlobalOptions_0.1.2
[10] zlibbioc_1.46.0	vctrs_0.6.3	memoise_2.0.1
[13] RCurl_1.98-1.12	MultiDataSet_1.28.0	ggtree_3.8.2
[16] askpass_1.2.0	rstatix_0.7.2	S4Arrays_1.2.0
[19] broom_1.0.5	gridGraphics_0.5-1	plyr_1.8.8
[22] cachem_1.0.8	qqman_0.1.9	igraph_1.5.1
[25] iterators_1.0.14	lifecycle_1.0.3	ropls_1.32.0
[28] pkgconfig_2.0.3	gson_0.1.0	Matrix_1.6-1
[31] R6_2.5.1	fastmap_1.1.1	clue_0.3-64
[34] GenomeInfoDbData_1.2.10	MatrixGenerics_1.12.3	digest_0.6.33
[37] aplot_0.2.0	enrichplot_1.21.2.001	colorspace_2.1-0
[40] patchwork_1.1.3	AnnotationDbi_1.62.2	S4Vectors_0.38.1

[43] RSpectra_0.16-1	GenomicRanges_1.52.0	RSQLite_2.3.1
[46] ggpubr_0.6.0	fansi_1.0.4	httr_1.4.7
[49] polyclip_1.10-4	abind_1.4-5	compiler_4.3.1
[52] doParallel_1.0.17	bit64_4.0.5	withr_2.5.0
[55] downloader_0.4	backports_1.4.1	BiocParallel_1.34.2
[58] carData_3.0-5	viridis_0.6.4	DBI_1.1.3
[61] ggforce_0.4.1	ggsignif_0.6.4	MASS_7.3-60
[64] openssl_2.1.0	DelayedArray_0.26.7	rjson_0.2.21
[67] HDO.db_0.99.1	tools_4.3.1	scatterpie_0.2.1
[70] ape_5.7-1	glue_1.6.2	nlme_3.1-163
[73] GOSemSim_2.27.2	shadowtext_0.1.2	grid_4.3.1
[76] cluster_2.1.4	reshape2_1.4.4	fgsea_1.27.1
[79] generics_0.1.3	gtable_0.3.4	tidyr_1.3.0

[82] data.table_1.14.8 tidygraph_1.2.3 car_3.1-2

[85] utf8_1.2.3 XVector_0.40.0 BiocGenerics_0.46.0

[88] foreach_1.5.2 ggrepel_0.9.3 pillar_1.9.0

[91] stringr_1.5.0 yulab.utils_0.0.9 limma_3.56.2

[94] circlize_0.4.15 splines_4.3.1 dplyr_1.1.3

[97] tweenr_2.0.2 treeio_1.24.3 lattice_0.21-8

[100] bit_4.0.5 tidyselect_1.2.0 ComplexHeatmap_2.16.0

[103] GO.db_3.17.0 Biostrings_2.68.1 gridExtra_2.3

[106] IRanges_2.34.1 SummarizedExperiment_1.30.2 stats4_4.3.1

[109] graphlayouts_1.0.0 Biobase_2.60.0 matrixStats_1.0.0

[112] stringi_1.7.12 lazyeval_0.2.2 ggfun_0.1.2

[115] codetools_0.2-19 ggraph_2.1.0 tibble_3.2.1

[118] qvalue_2.32.0 ggplotify_0.1.2 cli_3.6.1

[121] reticulate_1.31	munsell_0.5.0	Rcpp_1.0.11
[124] GenomeInfoDb_1.37.4	png_0.1-8	parallel_4.3.1
[127] ggplot2_3.4.3	blob_1.2.4	clusterProfiler_4.8.3
[130] calibrate_1.7.7	DOSE_3.26.1	bitops_1.0-7
[133] tidytree_0.4.5	viridisLite_0.4.2	scales_1.3.0
[136] purrr_1.0.2	crayon_1.5.2	GetoptLong_1.0.5
[139] rlang_1.1.1	cowplot_1.1.1	fastmatch_1.1-4
[142] KEGGREST_1.40.0		
